# The Roles of Signaling in Cytoskeletal Changes, Random Movement, Direction-Sensing and Polarization of Eukaryotic Cells

**DOI:** 10.3390/cells9061437

**Published:** 2020-06-10

**Authors:** Yougan Cheng, Bryan Felix, Hans G. Othmer

**Affiliations:** 1 Bristol Myers Squibb, Route 206 & Province Line Road, Princeton, NJ 08543, USA; yougan.cheng@gmail.com; 2 School of Mathematics, University of Minnesota, Minneapolis, MN 55445, USA; felix077@umn.edu

**Keywords:** cell motility, signal transduction, actin dynamics, intracellular waves, polarization, direction sensing, symmetry-breaking, biphasic responses, reaction-diffusion, membrane and cortical tension

## Abstract

Movement of cells and tissues is essential at various stages during the lifetime of an organism, including morphogenesis in early development, in the immune response to pathogens, and during wound-healing and tissue regeneration. Individual cells are able to move in a variety of microenvironments (MEs) (A glossary of the acronyms used herein is given at the end) by suitably adapting both their shape and how they transmit force to the ME, but how cells translate environmental signals into the forces that shape them and enable them to move is poorly understood. While many of the networks involved in signal detection, transduction and movement have been characterized, how intracellular signals control re-building of the cyctoskeleton to enable movement is not understood. In this review we discuss recent advances in our understanding of signal transduction networks related to direction-sensing and movement, and some of the problems that remain to be solved.

## 1. Introduction

Active movement of cells, either individually or collectively, is essential in early development, in the immune response, and in a variety of other processes [1]. Evolution has led to a number of different modes of active movement of single-cell organisms, ranging from crawling to swimming. Certain bacteria, such as *E. coli*, use flagella to swim, while paramecia use cilia, but each can only use one mode of movement. However, some motile eukaryotic cells are more flexible and can adopt the mode used to the environment in which they find themselves, swimming in some MEs and crawling in others [2]. In addition, whether cells move individually or collectively can depend on the density of the extracellular matrix (ECM) in their ME [3]. This plasticity or adaptability has significant implications for understanding cell motility for it implies that the focus must be on the behavior of the integrated system, not simply on its component parts.

Cells use complex signaling pathways to control the local structure of actin networks, which comprise both branched and linear filaments, and the contraction of myosin-II (myo-II) motors, which are embedded both in the cortical cytoskeleton—the cross-linked actin network that is linked to the membrane—and in the remaining intracellular network of actin filaments, microtubules and other structures. Together these networks, which we call the cytoskeleton (CSK), produce the forces that drive cell shape changes and movement, whether they are random in spacetime or spatially directed in response to signals in the environment. In order to move, the forces must be transmitted to the environment, and the ease or difficulty of movement in a given context produces feedback that is used by cells to control their movement. Understanding how signaling networks and the mechanical responses are integrated to produce shape changes and movement, and how external signals—either in the form of imposed spatially or temporally varying signals or those generated by movement—affect the responses, remains a major challenge in biology. The complexity of these processes is such that mathematical models are essential for synthesizing what is known to unify observations, and for making predictions that can guide further experimental work.

In this review, we focus on the processes that control single-cell motility, which we categorize as (i) signal transduction, (ii) actin network dynamics and intracellular waves, (iii) direction-sensing and polarization, and (iv) integration of signaling and structural changes. Each of these is a major topic in itself, and while individual processes have been reviewed elsewhere [4,5,6,7], our objective is to give a broader overview of their interdependence. We begin with a brief sketch of this that hints at how they are integrated.

In the absence of directional signals in their ME, many cell types, including neutrophils and *Dictyostelium discoideum* (Dicty), explore their environment more-or-less randomly [8,9], and therefore the intracellular signaling networks that control the shape changes must be tuned to produce signals that generate this movement. Thus, a first objective is to understand how the pathways that control actin network dynamics can produce random extensions of the membrane, whether in the form of filopodia, pseudopodia or lamellipodia. To this end, it is necessary to determine whether the known pathways can at least generate random actin waves that might trigger such protrusions, ignoring whether the membrane deformations needed for a protrusion emerge from these actin structures. Some have suggested that an integrated model for direction-sensing, adaptation, and signal-independent actin waves is comprised of two components—a signal-transduction excitable network (STEN) coupled to a CSK oscillatory network (CON) [10]. In Section 3 we review the signal-transduction networks in Dicty and neutrophils and discuss the dynamics of the Ras-PI3K-PTEN pathway. In Section 4 we discuss a number of models for actin waves that have been developed and show that a recent, detailed model of frustrated phagocytosis can replicate the experimentally observed waves in this system.

In the presence of a chemotactic, durotactic or other directionally biased signal in the environment the cells must orient or re-orient themselves appropriately, and this involves both direction-sensing and polarization. This is a two-step process, the former defined as determining the most favorable direction of movement, whether up the gradient of an attractant or down that of a repellent. This is a classical problem and it is well understood what a cell must do, and in Section 5.2 we describe a model for direction-sensing in Dicty that is based on extensive experimental data. The second step of the process is polarization—often referred to as symmetry-breaking [11]—in which the cell establishes an internal directional bias in the cytoskeletal structure. Simply put, this amounts to establishing a front and a back of a motile cell. However, polarization is not restricted to migrating cells—epithelial cells and budding yeast cells can become polarized without moving, the former to distinguish the ‘top’ from the ‘bottom’ and the latter to establish the budding site. The dynamics of the integrated signaling networks and their role in generating polarization in an external signal is discussed in Section 6.

## 2. The Primary Modes of Cell Movement

Since different types of cells use vastly different modes of movement that involve different modes of control of the CSK, we begin with a brief description of the various modes. An extended review of cell motility is given elsewhere [2].

**Figure 1:**
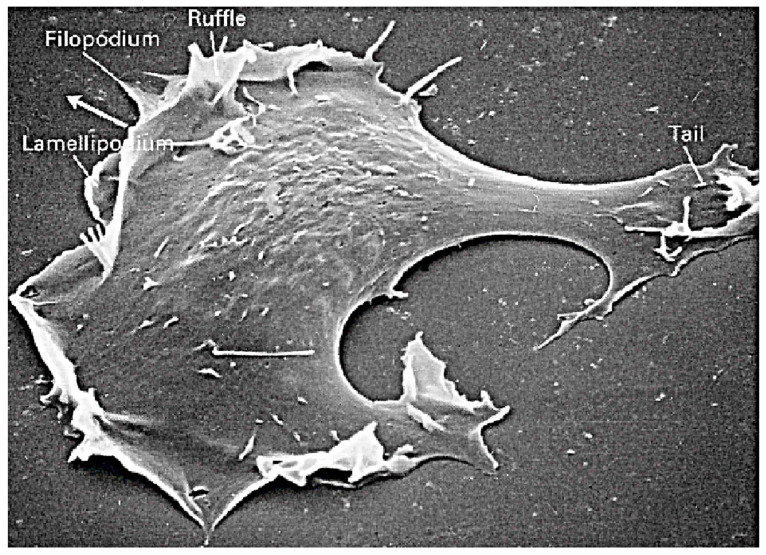
A fibroblast cell on a surface.

The two major modes of eukaryotic cell movement are called mesenchymal and amoeboid [12,13]. Mesenchymal movement is used by fibroblasts and various tumor cells, and usually involves strong adhesion to the substrate and extension of relatively flat lamellipodia at the leading edge (Figure 1). The construction of lamellipodia involves nucleation of filaments at the membrane that then treadmill as in solution. The densely branched structure of the network arises via Arp2/3-controlled nucleation of branches on existing filaments [14]. Transmission of force to the environment involves integrin-mediated focal adhesions that are connected to the CSK via stress fibers, and this mode often involves proteolysis of the ECM to create a pathway for the cell [15].

**Figure 2:**
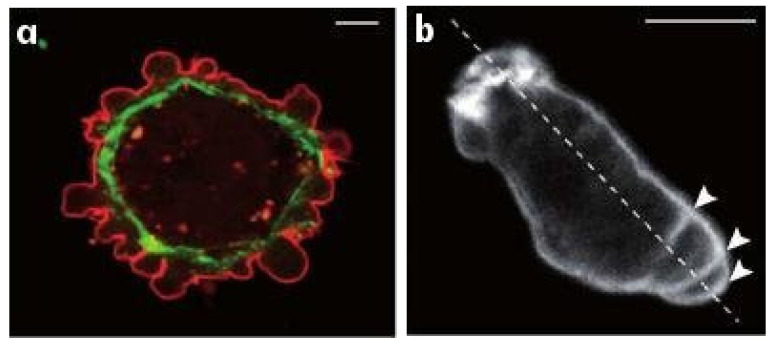
(**a**) Blebbing on a melanoma cell: myosin (green) localizes under the blebbing membrane (red) (**b**) The actin cortex of a Dicty cell migrating to the lower right. Arrowheads indicate the successive blebs and arcs of the actin cortex. The scale bar is 5 μm is each panel. From [26].

The amoeboid mode of movement is based on a less-structured CSK and typically involves less adhesion to the substrate. In the amoeboid mode cells adopt a more rounded cell shape and often have a highly contractile ‘tail’ called the uropod [16]. There are several distinct types of amoeboid motion that have been identified. In the first type cells generate a rearward flow of actin in the cortex, which leads to a reactive tension gradient in the membrane that propels the cell forward. This is called the tension- or friction-driven mode [17]. In the second type cells use actin-rich protrusions called pseudopodia at the leading edge, or by blebbing, in which cycles of extension of the front and retraction of the rear as shown in Figure 2b are used. In a third mode Dicty cells on a substrate move by deforming their shape in a wave-like manner [18], similar to a swimming mode described below.

In an environment less favorable to mesenchymal movement, eg., due to changes in the stiffness or adhesiveness of the substrate [19,20], cells compensate by undergoing a ‘mesenchymal-to-amoeboid’ transition (MAT) [21,22]. For example, leukocytes can use the mesenchymal mode when moving in a tissue, but can also migrate in vivo without using integrins, instead using a ‘flowing and squeezing’ mechanism [16]. In a cyclic AMP (cAMP) gradient on a rigid substrate, Dicty moves either by extending pseudopodia or by blebbing, and determines which mode to use by monitoring the stiffness of the surroundings. Pseudopodia are used in a compliant medium and blebbing is used in stiffer media [23]. While pseudopods and blebs involve very different actin dynamics, with the former based on a highly branched dendritic network, whereas the latter involves high contractility of the cortex that produces a high intracellular pressure and detachment of the membrane from the cortex at the leading edge, they can coexist at the leading edge [23]. Furthermore, blebs may transform into pseudopods by continued actin polymerization at the cortex, while pseudopods can spawn blebs at their edges [23,24]. Thus there can be a delicate balance between them. Finally, some cells move only by blebbing. Certain types of carcinoma cells are immobile on 2D substrates, but polarize spontaneously, form blebs, and move efficiently in a confined environment [25].

**Figure 3:**
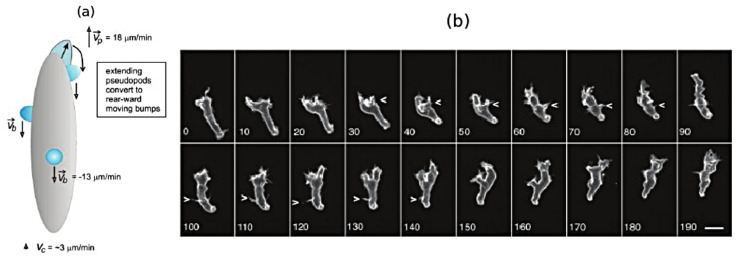
How Dicty amoebae swim by protrusions and shape changes. (**a**) a schematics of a swimming cell with 3 protrusions. From [31]; (**b**) the shape of a Dicty cell as it swims. Scale bar 10 μm. From [27].

While it is less frequently used as a mode of movement, Dicty and neutrophils can swim in a fluid [27], and presumably use this mode to move through fluid-filled voids in their environment. Figure 3a shows a schematic that illustrates how Dicty moves by propagating protrusions down its length, and Figure 3b shows a time sequence of shape changes that Dicty executes as it swims toward the site of an attractant. This has been modeled and analyzed [28], and it was shown how characteristics of the protrusions, such as their height, affect the swimmer’s speed and efficiency. In addition, it is also known that Dicty cells can swim for several cell lengths without shape changes [29], and it has been shown that they can do so by creating an axial tension gradient in the membrane [17].

Swimming and crawling are two very different strategies for movement, and raise the problem of understanding how mechano-chemical sensing of the environment and transduction of the information to the intracellular networks is used to control the structure of the CSK [30], which is clearly different in a fibroblast from that in a swimming cell. Protrusions and other shape changes require forces that must be correctly orchestrated in space and time to produce net motion—those on cells in Figure 2a are not, while those in Figure 2b are—and to understand this orchestration one must couple the intracellular dynamics with the state of the surrounding fluid or ECM. Tension in and curvature of the membrane and cortex have emerged as important determinants in the orchestration, whether in the context of undirected cell movement, or in movement in response to environmental cues [17].

Finally, since cells can be motile in the absence of extracellular signals, the autonomous dynamics of the actin network governing un-stimulated movement must be understood separately from the stimulated response. The fact that different modes can coexist in cells such as Dicty suggests that the balance between factors or pathways that determine the modes may be delicate.

## 3. The Signal-Transduction Network in Eukaryotic Cells

Prokaryotes such as *E. coli* are too small to measure the spatial gradient of signals across their body length, and thus developed a ‘run-and-tumble’ strategy in which they execute a random walk with persistence when searching for a favorable environment or trying to leave an unfavorable one. To implement this strategy *E. coli* has developed a sophisticated signal-transduction network that controls the rotational bias of flagella that propel the cell [32,33]. Motile eukaryotic cells such as neutrophils, fibroblasts and Dicty have also developed search strategies that involve execution of a persistent random walk [8,9], but since they are large enough to measure signal differences over their body length, the mechanism for implementing the search strategy is quite different. For instance, cAMP is a chemoattractant for Dicty, but in the absence of an external signal, cells spontaneously form and extend pseudopods [34,35,36], which involves localized re-building of the actin network. These new pseudopodia can either be retracted or can attach to the substrate, and in the latter case the cell adopts a polarized shape and moves in the new direction with a persistence time of about 9 min [37]. Of course the question is which intracellular signaling pathways control the location of a new pseudopod and the remodeling of the cortex and CSK to form a pseudopod, and how is this system biased by an external signal. Here the current state of knowledge for eukaryotes is far behind that in *E. coli*. Due to its genetic and biochemical tractability, Dicty is a widely used model system for studying these questions, and is to date the best understood eukaryotic system [38,39].

### 3.1. The Signal-Transduction Networks in Dictyostelium and Neutrophils

**Figure 4:**
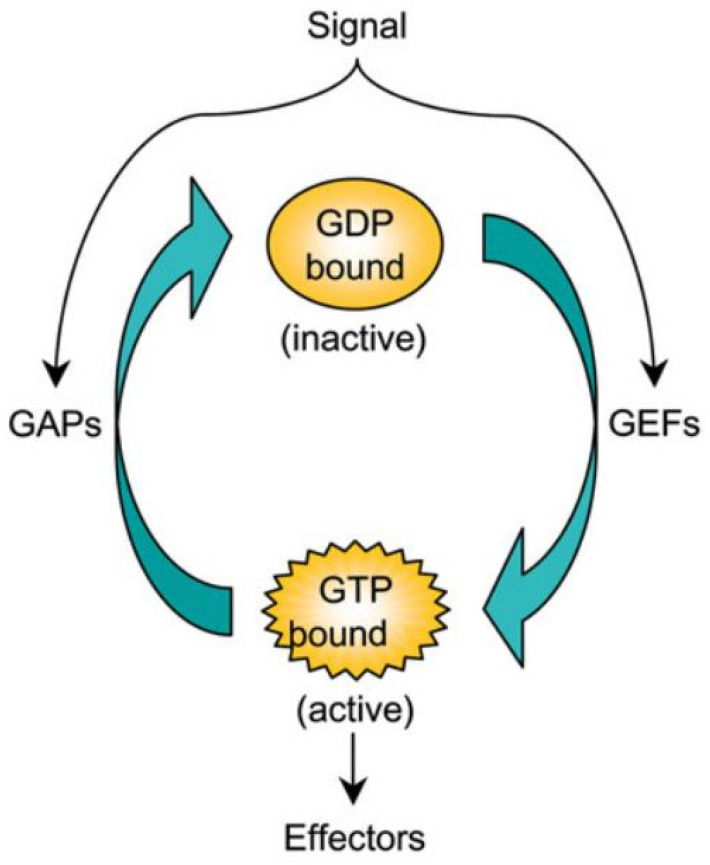
The molecular switch for a RHo GTPase—Rho is ‘on’ or active when GTP-bound, and ‘off’ or inactive when GDP-bound. From Charest et al. [43].

The small GTPases in the Ras superfamily, of which there are 150 human members and orthologs in yeast, Dicty and other species [40], are essential components of the pathways controlling the CSK in eukaryotic cells. These are grouped into five families—Ras, Rho, Rab, Ran and Arf—of which the first two are of primary interest here. The GTPases act as molecular Boolean switches in signaling pathways, with the on-off state determined by whether they are GTP-bound (‘on’) or GDP-bound (‘off’). The binding state is controlled by GEFs (GTP exchange factors) or GAPs (GTPase-activating factors (Figure 4). The state of the switch can be controlled by controlling the GEFs and GAPs, which in turn can be controlled by other factors, and thus there are at least a two levels of control involved. Active GTPases act on downstream effectors to control network structure and dynamics by controlling two classes of actin nucleators, WASP and SCAR/WAVE proteins in one and Diaphanous-related formins in the other. The first controls production of branched dendritic networks, and the other long, frequently bundled, linear filaments. By controlling their localization with membranes in the presence of different signals, the spatial location of different network types can be controlled in a cell.

**Figure 5:**
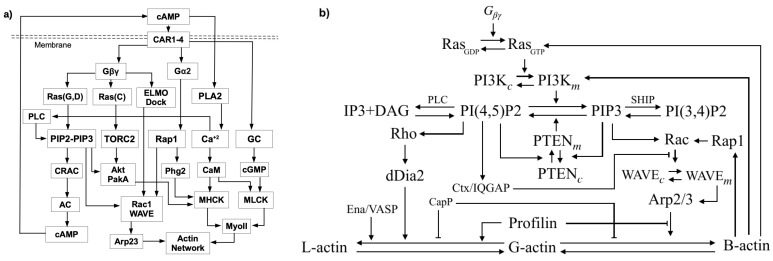
(**a**) The principal pathways in cAMP signal transduction in Dicty. CAR1-4: the cAMP receptors, $G_{\alpha_{2}}$ and $G_{\beta \gamma }$: components of the G-protein used for the transduction of the cAMP signal, Ras, Rac1: small GTPases, PIP2 and PIP3; membrane components that are inter-converted via phosphorylation and de-phosphorylation, ${\mathrm{IP}}_{3}$ and DAG: products of PIP2 degradation, GC: guanylate cyclase—the enzyme that produces cyclic GMP (cGMP), AC: adenylate cyclase—the enzyme that produces cAMP, Myo-II: a motor protein involved in contraction of the actin network. Arrows indicate an effect, but not whether it is activating or inhibiting, and feedback steps are not shown. (**b**) Details of the PIP2-PIP3 subnetwork.

Three Rho GTPases—Rho, Rac and Cdc42—all activated by Ras, control three pathways in neutrophils that control actin network contraction, extension of filopodia [41], and lamellipodia [15,42], resp. Cdc42 and Rac control dendritic network formation by activation of scaffold proteins of the WASP family, which when activated facilitate actin polymerization by regulating Arp2/3 [43]. When activated, RhoA facilitates formation of actin bundles and stress fibers by activating the contraction of myo-II, which is done by deactivating MLCPase, an inhibitor of myosin contraction [44]. A sketch of these pathways in Dicty, which lacks both Rho and Cdc42, but uses Akt/PKB instead, is shown in Figure 5. Figure 5a shows the five main pathways in Dicty that are involved in transducing an extracellular cAMP signal to changes in the actin network.

The first step is binding of cAMP to one of the G-protein-coupled cAR receptors, which activates the G-proteins. G-proteins consist of an α subunit that contains a GTP/GDP binding domain as well as intrinsic GTPase activity, and a complex of a β and a γ subunit. The α and βγ subunits dissociate after activation, and each can activate downstream signaling pathways as shown in Figure 5a. A major one is via $G_{\beta \gamma }$, RasG,D and PI(4,5)P2/PI(3,4,5)P3 (PIP2/PIP3), to Rac1, adenylate cyclase and cAMP, another is via Ras C and the TOR pathway, also to Rac1, and other pathways are driven by PLA2, by guanylate cyclase (GC), and by ${\mathrm{Ca}}^{+2}$. While many components are shown there, the diagram only contains representatives of the principal actors and pathways. For example, there are a number of $G_{\alpha }$s, and five different Ras proteins, three of which, RasG, Ras D and RasC are shown and are principals in the chemotaxis pathways. RasG is a primary regulator via localization of phosphatidylinositol-3 kinase (PI3K), which converts PIP2 into PIP3, while RasC regulates activity of the target of rapamycin complex 2 (TORC2), a parallel pathway that regulates chemotaxis. Figure 5b shows an expanded version of the PIP2-PIP3 component, which is central to the waves described later [45,46]. A mechanistic description of the PLC and CRAC pathways is given elsewhere [46,47].

Assembly of the motor protein myo-II is controlled in part by PAKa via its effect on MHCK [48], and contraction is stimulated via the cGMP pathway by deactivation of an inhibitor of myo-II contraction [44]. The balance between the Rac1, Rap1 and GC pathways, in conjunction with other factors such as membrane tension, determine whether dendritic network formation (B-actin) or bundling of long filaments (L-actin) and myo-II-controlled contraction dominates, and as will be seen later the competition between them can lead to complex patterns of traveling waves.

Not all steps are shown in Figure 5, and other feedback interactions will be discussed later. Mutual inhibition between these pathways may ensure that the mesenchymal and amoeboid modes are mutually exclusive in some cells, but it is not absolute, since Dicty can use a mixed-mode strategy that involves either pseudopodia or blebbing [24]. High-level models of some of the interactions shown in Figure 5 are reviewed in [49,50,51].

### 3.2. The Dynamics of the Ras-PI3K-PTEN Pathway

**Figure 6:**
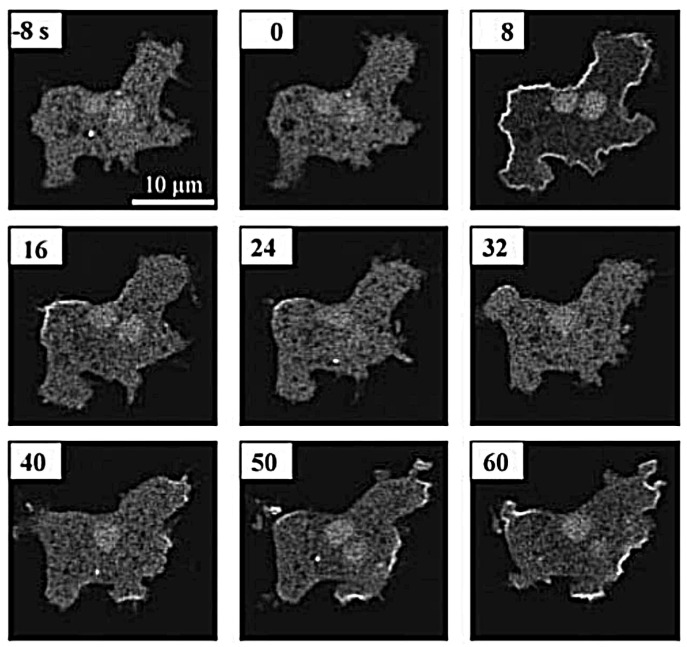
GFP in the cytosol before cAMP stimulation, a transient translocation of GFP to the entire boundary of the cell at 8 s after addition of cAMP, and patches of GFP at the boundary after 40 s. From Postma et al. [53].

In the absence of cAMP stimuli Dicty cells plated on glass extend pseudopods in random directions, but under spatially uniform cAMP stimuli aggregation-competent cells first respond by suppressing existing pseudopods and rounding up (the ‘cringe response’), which occurs within about 20 s and lasts about 30 s [52]. This first phase of the response is characterized by uniform and transient membrane localization of ${\mathrm{PH}}_{CRAC}$-GFP, a marker for PIP3, along the cell periphery within 10 s [53,54]. This fast phase of PIP3 accumulation is less affected by PI3K inhibition, which suggests that another pathway may be involved. Given that ElmoE interacts directly with $G_{\beta \gamma }$ [55], one could speculate that ElmoE might be essential in the first phase rise that occurs on a faster time-scale. Under uniform stimuli there is a second phase characterized by localized patches of labeled CRAC (Figure 6), extension of pseudopods in various directions, and an increase in the motility [56,57,58]. The second-phase rise is probably due to other signaling pathways, possibly involving F-actin (actin filaments of either type), that react on a slower time-scale. A localized application of cAMP elicits the cringe response followed by a localized extension of a pseudopod near the point of application of the stimulus [59].

A model described in Section 5.2 shows how the cell can use Ras activation to determine the direction in which the signal is largest, but how it organizes the motile machinery to polarize and move in that direction is still a major question from both the experimental and theoretical viewpoint. A subsequent step downstream of Ras is the generation of pleckstrin homology (PH) binding sites by the phosphorylation of the membrane lipid PIP2 by phosphoinositide 3-kinases (PI3Ks) to produce PIP3, which in turn is dephosphorylated to produce PtdIns(3,4)P2 (Figure 5b). In Dicty, PIP3 is produced by a class IA type kinase (PI3K1 and PI3K2) and a class IB type, kinase designated PI3$K_{\gamma }$ [60,61]. The former are activated via cytosolic tyrosine kinases, and thus may contribute to basal activity, whereas the latter consists of a catalytic unit and binds to F-actin via the N-terminal region. The latter fact may explain why the fast phase of the response to a uniform stimulus is PI3K insensitive. Both PIP3 and PI(3,4)P2 provide binding sites for various cytosolic proteins containing PH domains (PHPs) and recruitment is rapid: localization of PHPs at the membrane peaks 5–6 s after global stimulation with cAMP [60,62]. Both PIP3 and PI(3,4)P2 are tightly regulated by the phosphatases PTEN and SHIP, and within 10–15 s following uniform cAMP increases the PHPs return to the cytoplasm [60,63]. This rapid increase of PIP3 at the membrane couples the extracellular signal to actin polymerization via Rac1-WAVE-Arp2/3 (Figure 5a), which creates a feedback loop that leads to increased PI3K binding and increased PIP3 production. The level of activated G-proteins in continuously stimulated cells reaches a stimulus-dependent level, while membrane-associated CRAC first increases, but then returns to its basal level. Therefore, adaptation of the PIP3 and cAMP responses, as well as directional sensing, is downstream of $G_{\beta \gamma }$ and upstream of PIP3 and CRAC [64,65].

The ratio of PIP2 to PIP3 has a significant role in the blebbing vs. pseudopod dichotomy mentioned earlier. This ratio dictates if either detachment of the membrane from the cortex or, B-actin formation at the membrane occurs. A reduction of PIP2 increases blebbing, possibly via its effect on membrane-cortex adhesion [66], whereas the absence of PIP2 conversion in *pi3$k^{-}$* leads to greatly reduced production of blebs compared with wild-type cells [23,24]. One can see in Figure 5 that another balance, that between the Ras-independent and Ras-dependent pathways may be an essential factor in resolving the blebbing-pseudopod competition.

## 4. Intracellular Actin Waves in the Absence of Directional Signals

As remarked earlier, cells can execute a persistent random walk in a signal-free environment, and it has been found that the intracellular components of the network exhibit a variety of spatio-temporal wave patterns under such conditions. The first to observe actin waves in Dicty were Vicker et al. [67,68,69], and Vicker [70] was the first to suggest that these waves were generated by an excitable reaction-diffusion system involving actin dynamics. Since then such waves have been observed in Dicty, neutrophils and other cell types [8,9,71,72,73,74,75,76,77]. Inagaki et al. [78] provide a broad overview of waves and their role in various aspects of cell dynamics.

**Figure 7:**
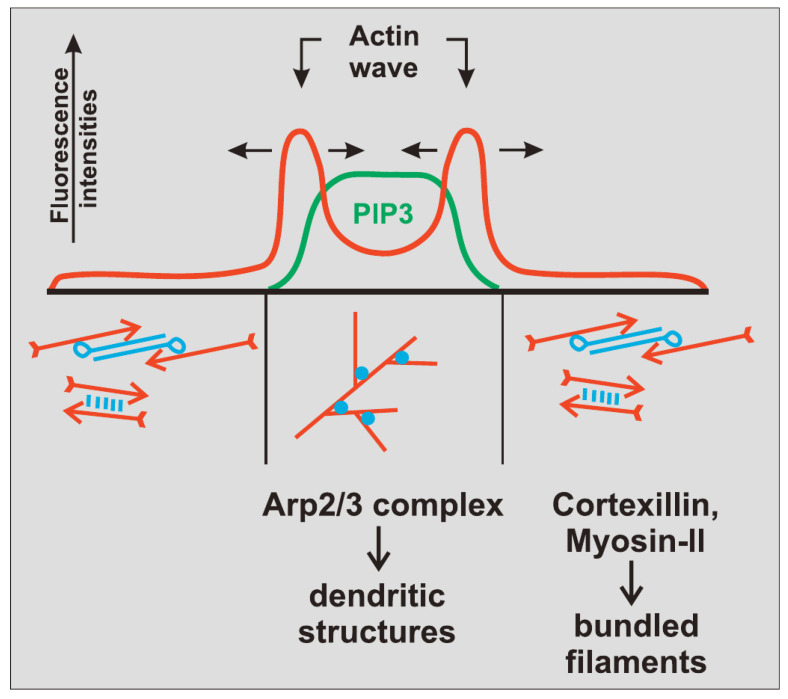
A cross-section of the waves in LatA-treated Dicty cells. From Gerisch [79].

The actin waves in Dicty and macrophages arise during re-building of the actin network following treatment with latrunculin A (LatA), which sequesters G-actin and leads to disintegration of the network and rounding of the cells. After removal of the drug the cells return to their pre-stimulus state, but in the interim there are distinct domains of the membrane that is in contact with the surface in which different actin structures exist (Figure 7). In one PIP3, Ras and Arp2/3 are high and the network is dendritic, whereas in the other PIP3 is low, PIP2 and cortexillin are high, and F-actin is is linear and bundled. The existence of two distinct domains separated by a propagating actin wave suggests that the underlying dynamics are bistable, with one state in which PIP3 is high and PIP2 is low, and the other in which the roles are reversed. The waves that exist between domains of high and low PIP3 are usually closed and of varying shape, and actin recovery after bleaching shows that they propagate by treadmilling [79]. Myosin-IB, a membrane-cortex linker protein [80,81], is found at the front of a wave, and a dense dendritic network is found in the high PIP3 domain. Other components that are found where PIP3 is low include coronin, which inhibits filament nucleation and indirectly regulates cofilin activity via dephosphorylation [82], and cortexillin, which organizes actin filaments into anti-parallel bundles.

**Figure 8:**
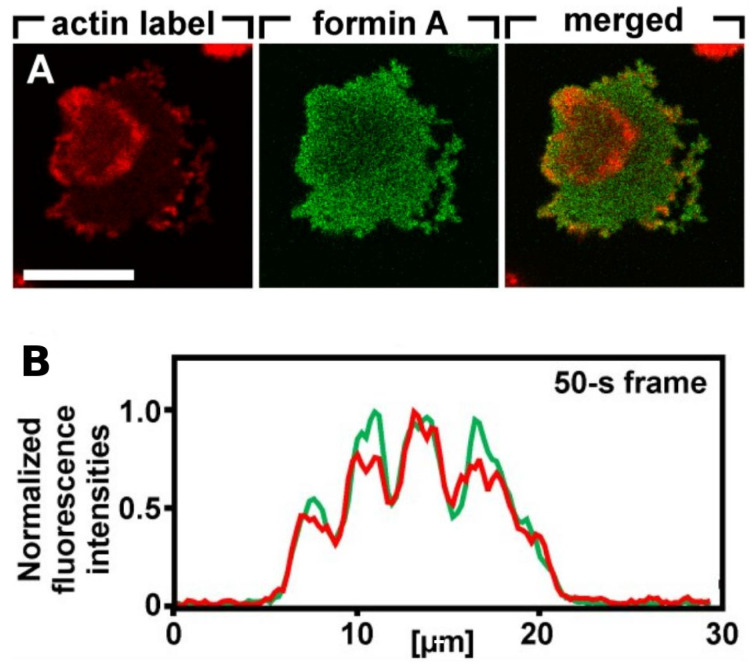
(**A**) Individual and overlay images of actin and formin A in a cell recovering from treatment with 5 μM latrunculin, showing actin and formins on the substrate-attached cell surface. Scale bar 10 μm. (**B**) The spatial distribution of formin (green) and Arp2/3 (red) along the wavefront. From Ecke et al. [83].

Because it is the balance between the Rac1, Rap1 and GC pathways that determines whether formation of dendritic networks or formation of linear actin dominates, experimentalists believed that the complex patterns of traveling actin waves in the cortex that are observed in the absence of directional signals may be the result of competition between them. Figure 7 shows that Arp2/3 is prevalent in the inner region while bundled filaments are dominant in the outer region, but the dichotomy may not be so clean. Recent work shows that formins, which nucleate and elongate actin filaments, are distributed throughout the inner and outer regions, but the type of formin varies, and the waves disappear when cells are treated with a formin inhibitor [83]. Figure 8A shows that formin A is high outside the wave, reduced in the inner region, but high in the wave front and back. Figure 8B shows the coexistence of formin B (green) and Arp2/3 (red) along the wave front. Thus the actin wave is undoubtedly a mix of long filaments and branched network. This changes the picture of how wave formation is controlled significantly, since it indicates that the formin-controlled pathway is essential.

Oscillations in the local network dynamics, as well as waves in the corresponding reaction-diffusion systems, often originate from a certain balance between positive feedback and slow inhibition in the network. The simplest mathematical model that can produce the different wave types leads to the following equation, which stems from reaction and diffusion in a two-phase medium with rapid interphase transport.

(1) $$\frac{\partial u}{\partial t}+\left(f_{0}+f_{1}u\right)\frac{\partial u}{\partial x}=\frac{\partial^{2}u}{\partial x^{2}}+g\left(u\right)$$

The second term on the left side represents an active or convective transport process in the fluid phase and g(u) is qualitatively a cubic nonlinearity with zeroes $u_{1}\leq u_{2}\leq u_{3}$. By adjusting the parameters $f_{0}$ and $f_{1}$ one can obtain a propagating transition wave from $u_{1}\to u_{3}$ or $u_{3}\to u_{1}$, periodic and damped oscillatory waves, or transition waves [84]. Furthermore, if a second mechanism controls one of these parameters, one can make the waves stall, as in [85] or reverse the waves at the boundary. The transition waves are stable on the line, while the others are not, but this is not relevant to the scale of a cell. However, this is only a cartoon description—the underlying mechanism is much more complicated.

While the observed patterns of wave initiation and propagation suggest that the waves are governed by an excitable system, it has been difficult to identify a minimal set of components of the network shown in Figure 5 responsible for them. For example, it is known that the Ras activation step in isolation is not excitable—it responds proportionately to any stimulus and adapts, and thus if Ras is part of an excitable STEN there must be downstream feedback on Ras. However, Ras and PI3K can still be activated in $g_{\beta \gamma }$-null cells, thus eliminating the effect of extracellular cAMP [34]. These authors and others [86] suggest that there is a feedback loop from F-actin to Ras, as shown in Figure 5b, but the feedback may arise from components further up the pathway. Another question stems from the fact that it is often difficult to obtain the long-term evolution of the waves in order to determine whether they are transition waves between two distinct steady states, or pulse waves that begin and end at the same steady state. A model of frustrated phagocytosis described later supports both types in different parameter regimes [87].

### 4.1. Models of Intracellular Waves in Dicty

It was reported that Ras waves exist in un-stimulated Dicty cells in the absence of both PI3K and F-actin feedback, and it was concluded that Ras waves drive PIP3 waves [88]. The authors suggest that spontaneous Ras wave formation is possible without any downstream feedback and that this drives PI3K waves, and they develop a model in which there is feedback between the RasGTP and RasGAP to obtain waves. Computations using a model described in Section 5.2 show that without such feedback Ras alone cannot generate waves.

**Figure 9:**
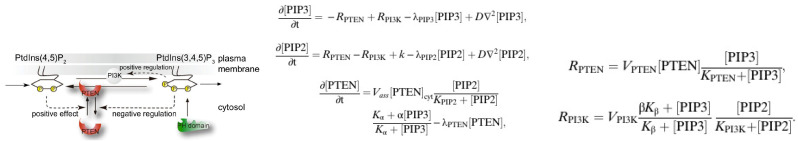
The model of the PIP2-PIP3 reaction and the governing equations. From Arai et al. [73].

**Figure 10:**
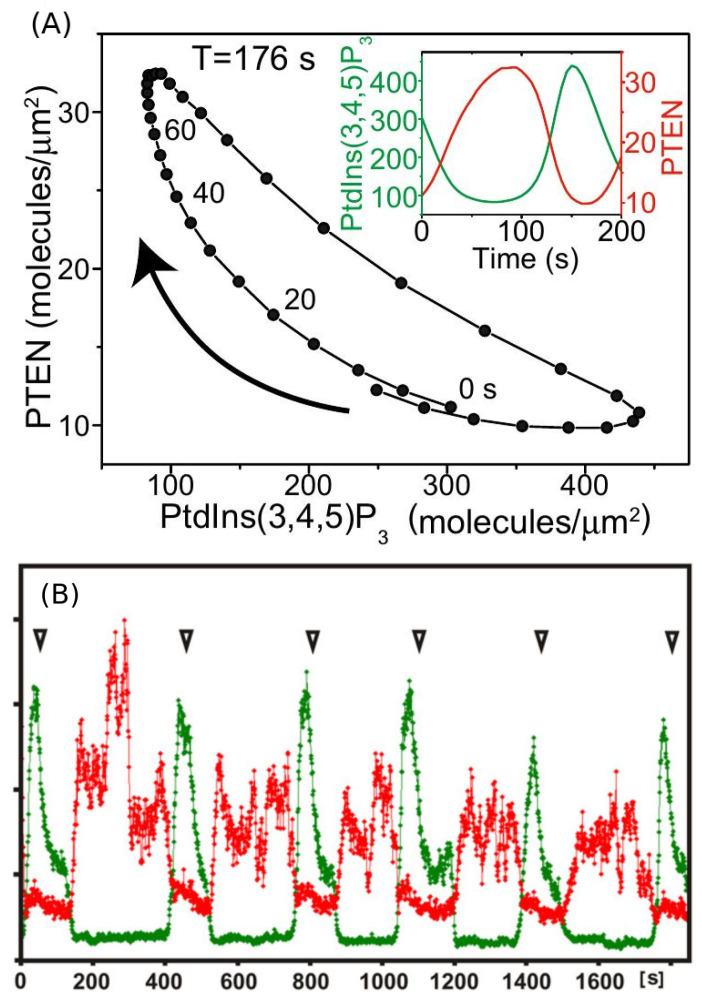
(**A**) The predicted PIP3-PTEN phase plane. From Arai et al. [73]. (**B**) A plot of PTEN (red) and PIP3 (green) vs. time taken in a small spot on the membrane. From Gerisch et al. [92].

An important feature of the waves is that typically, but not always, the domains described above are well separated, in that PIP3 is low where PIP2 is high and vice versa. There are several models proposed to explain the PIP3/PTEN dynamics [73,89,90], in which the reduction in PTEN/PIP2 plays the main inhibitory role such that the scheme follows the “substrate-depletion”-type mechanism in line with the fact that PIP3 and PTEN appear to be anti-phase. The authors find that F-actin is not required to generate PIP3/PTEN waves and propose a model based on the mechanism shown in Figure 9. The computational results shown in Figure 10 show the out-of-phase relationship found in some, but not all experiments. However, there are several concerns about the model. Concerning the effect of effect of PIP3 on PTEN, it has been pointed out that neither an increase in PIP3 nor a decrease in PIP3 levels influenced the membrane-binding of PTEN [91]. In addition, the sampling techniques may lead to erroneous conclusions. Gerisch et al. [92] show that when PIP3 and PTEN are sampled over the entire attached surface the results appear to be consistent with the anti-phase conclusion, but when sampled in a very small region the results are different. Figure 10B shows that there are regions in which PIP3 decreases sharply even as PTEN continues to decrease, which is contrary to the model predictions in Figure 10A. The switch from rise to fall of PIP3 is therefore unlikely to be caused by a depletion of PTEN, and the authors suggest that other factors are involved in the PIP3-PTEN dynamics. The model proposes that PIP3 might negatively regulate PTEN recruitment and positively regulate PI3K recruitment [73,90], but this has not been experimentally confirmed. It has been observed that PIP3 regulation of PI3K recruitment is F-actin dependent, and that there is no PI3K recruitment in LatA-treated cells [93,94].

One of those additional factors may be the actin network, for it is well established that the membrane-binding and activation of PI3-kinases depends on F-actin [93]. However, the role of actin or actin waves in the generation of the PIP3/PTEN patterns is still controversial. It is reported that PIP3/PTEN patterns disappear at a higher dose of LatA treatment (10 μM) [75,90], while PIP3 waves can still be observed in mild LatA treatments (0.5–2.0 μM). Nishikawa et al. [75] also reported that PIP3 waves reappear with addition of 1 nM cAMP under 10 μM LatA treatment. On the other hand, Arai et al. [73] report that PIP3/PTEN patterns are formed in the presence of 5 μM latrunculin A, a concentration that the authors considered to be sufficient for the complete inhibition of actin polymerization.

Another unknown in establishing a network concerns other factors affecting PIP2. In addition to the PTEN-regulated supply from PIP3, PIP2 can be supplied by PTEN-independent pathways [87,95,96,97], and as shown in Figure 5b, PIP2 can be degraded through calcium-dependent PLC activity and via PI3K- and PLC-independent pathways [4,95]. Moreover, PTEN interacts with lipids through several binding and catalytic domains [98,99,100,101,102], and it has been proposed that positively charged residues in the PIP2-binding and C2 domains can recruit PTEN to the plasma membrane through associations with negatively charged membrane lipid head groups [102,103,104,105,106], which suggests that there is a positive feedback loop in the PIP2-PTEN interaction.

**Figure 11:**
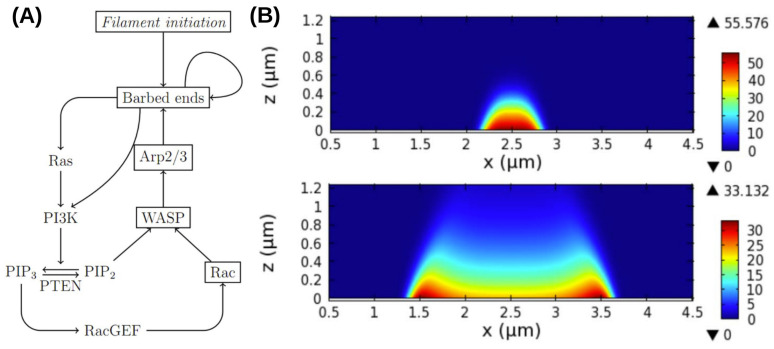
(**A**) A schematic of the network structure and molecular interactions in the model. (**B**) Two snapshots in time of an actin wave initiated at x = 2.5, showing the network density (color) as a function of space (*x*-axis) and network height (*z*-axis). From Khamviwath et al. [107].

A more detailed model of the observed waves that incorporates more molecular details of the PI3K pathway and the actin network dynamics has been proposed by Khamviwath et al. [107] (Figure 11A). Stimulation with a localized pulse of activated receptors, leads to a a single pulse, whose magnitude grows in time while the pulse spreads in both the x- and z-directions, the latter representing the height of the actin network (Figure 11B). A threshold stimulus is needed for initiation of a wave because the uniform rest state is stable, and this suggests that the model is excitable. The amplitude in the center decays later, and the pulse splits into two symmetric pulses, which is consistent with experimental observations [71].

Furthermore, Rac, which is a proxy for PIP3 in the model, shows no peaks, which is also consistent with the experimental observations [71]. Another prediction of the model is that the inclusion of PTEN leads to reversal of the waves, which agrees with the observations that the waves often propagate to the cell edge and then reverse direction. Other wave models are reviewed elsewhere [51], but much remains to be done to understand the internal structure of waves. For example, it has been found in macrophages described later that PIP(3,4)2 is enriched in the wave center, rather than PIP3, as in Dicty [76].

While the foregoing models involve PTEN and other components of the signaling network, it has been shown that SCAR/WAVE, Arp 2/3 and actin-binding proteins can generate rapid, localized oscillatory SCAR/WAVE-actin foci in Dicty cells lacking $G_{\beta }$ and PTEN [10], and more recently it was shown that $G_{\beta \gamma }$ has important effects on the dynamics. Knockout of $G_{\beta \gamma }$ completely blocks chemotaxis and CSK dynamics [4,34], but recently a $G_{\beta }$ sequestration technique to study the effect of $G_{\beta }$ on the spatial interaction of the foci was developed [108]. It was found that sequestration of $G_{\beta }$ induces large-scale oscillations of LimE-GFP, a reporter for F-actin, due to long-range coupling of actin foci, and that very few $G_{\beta }$-null cells display LimE-GFP oscillations. The global coupling of the local oscillators interferes with the sensing of extracellular signals and the changes in local actin dynamics needed to produce protrusions, but how this is effected remains to be explained.

### 4.2. Intracellular Waves in Frustrated Phagocytosis

Another system in which waves are observed involves macrophages that are in contact with a surface, undergoing a process called ’frustrated phagocytosis. Phagocytosis is a process in which phagocytes such as lymphocytes or macrophages engulf and destroy foreign bodies called pathogens in a tissue, and it is initiated when a cell of the immune system detects antibodies carried by a pathogen via receptors in the membrane. Signaling mechanisms that control the changes of the cellular cytoskeleton needed for engulfment of the pathogen lead to large mechanical deformations of the cell, and thus a mathematical model of the entire process would be extremely complicated. Recent experiments have used an experimental technique similar that used in LatA-treated Dicty cells in which the membrane does not deform, but rather, signaling triggers actin waves that propagate along the boundary of the cell [76].

**Figure 12:**
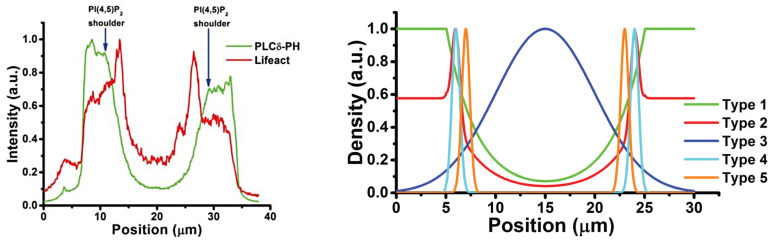
Left: A cross-section of an actin wave, showing actin (red) and PIP2 (green). The drop in PIP2 occurs at the boundary of the cell. Right: The spatial profiles of components in the wave, all normalized to their maximum value in the wave. Components were classified into 5 types, based on their pattern of in the waves: Type 1—Cortical actin, PI(4,5)P2, PI5K; Type 2—Total F-actin; Type 3—PI(3,4)P2, DAG; Type 4—Branched actin, N-WASP Type 5–SHIP2. Please note that PIP2 is constant in front of the wave. Taken from Masters et al. [76].

This eliminates the large-scale deformations and facilitates modeling of the wave dynamics. A model of the actin dynamics observed in frustrated phagocytosis that can replicate the experimental observations has been developed [87], and the key components that control the actin waves have been identified. Figure 12 shows the relative positions of different components in the wave found experimentally [76], and these should be compared with those shown for actin waves in Dicty in Figure 7.

**Figure 13:**
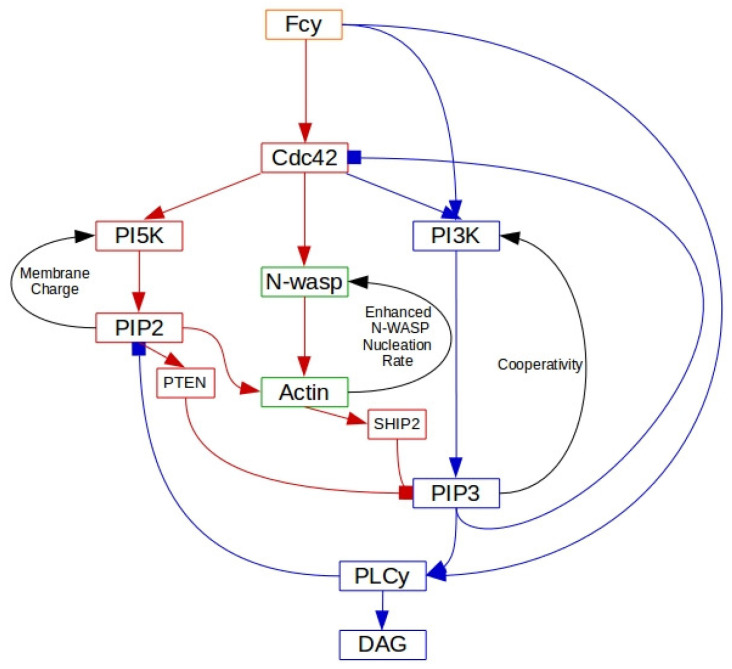
The signal-transduction steps following FcγR activation. From Ponce de Leon & Othmer [87].

**Figure 14:**
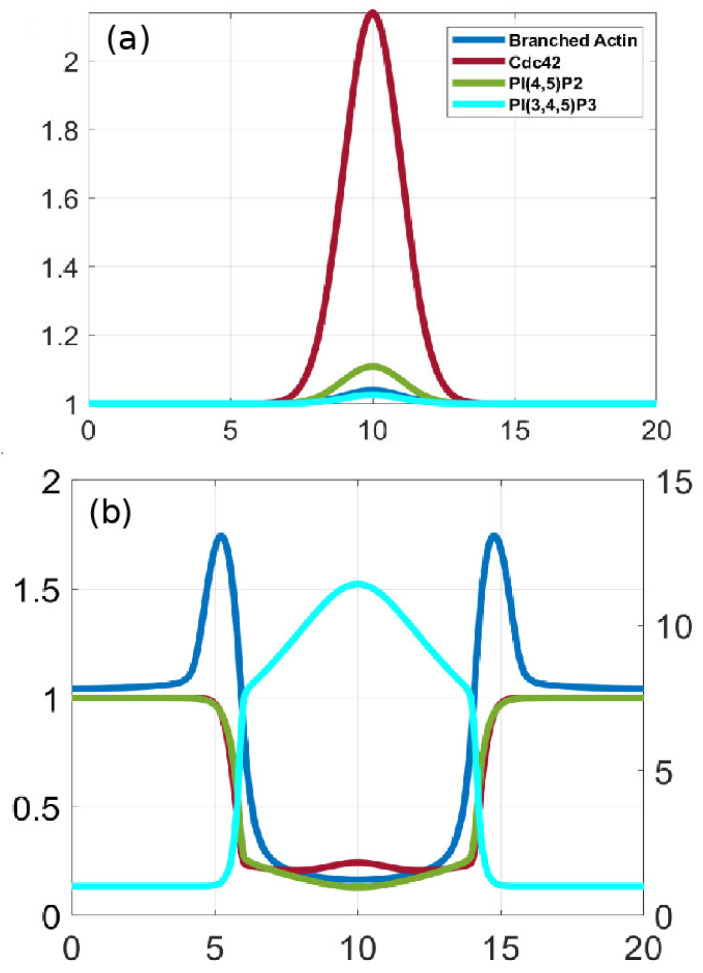
Branched actin wave initialization and propagation. Shown are the profiles of branched actin, PI(4,5)P2, Cdc42, and PIP3 at t=5 s (**a**), and 180 s (**b**) after a perturbation. The concentrations are normalized by the original steady state. The right axis on (**b**) is for PI(3,4)P2, which is shown in light cyan.

The signaling network is controlled by the FcγR receptor, and it is known that receptor activation following binding of the antibody immunoglobulin leads to a sequence of spatial and temporal changes in phosphoinositides, Rho-family GTPases and actin nucleation-promoting factors [109]. The spatio-temporal dynamics of these molecules control processes such as remodeling of the cytoskeleton, membrane fusion and the production of reactive oxygen intermediates that are necessary for particle internalization. However, it is not clear how the molecular scale activation of FcγR’s leads to the observed micron scale patterns of activation and inactivation of network components reflected in the propagating actin waves, and the network shown in Figure 13 was developed to address this issue [87]. Only membrane-localized components are shown, and all are placed in their approximate order of activation, with red faster than blue. The internal structure of the wave is captured in the model, and two snapshots of the time-evolution following a stimulus are shown in Figure 14.

There are a number of interesting predictions that emerge from the model. In particular, one is that diffusion coefficients of membrane-bound species must be larger behind the wavefront than in front to replicate the internal structure of the waves.

## 5. Models for Polarization and Direction Sensing

Cells in vivo are never either spatially homogeneous or geometrically symmetric at the molecular level, and thus the commonly used term *polarized* must be defined more loosely. Usually cell polarity refers to a spatial distribution of a protein, a lipid, the CSK, or another component that exhibits an identifiable spatial gradient. The ability to polarize at the cellular level is an essential property for cell division, cell-cell interactions such as mating in yeast cells, and the ability to move in a favorable direction or against an unfavorable one, and most cell types exhibit some form of polarity, which enables them to carry out these specialized functions. Polarity is very dynamic and can be very short-lived, as in the signaling patches that may generate actin waves and lead to small local membrane deformations, or longer-lived, such as the morphological polarity visible in cells such as neurons, in migrating fibroblasts, or in epithelial cells, which have a well-defined apical-basal polarity [110]. Given that polarity may be evanescent or persistent, a major question is how these spatio-temporal events are generated, and what distinguishes those that are evanescent from those that are imprinted for a longer time. For example, the actin patches described earlier are generally short-lived, and what determines the threshold that distinguishes a short-lived patch from a new pseudopod is not known. In the context of the problems described herein, the imprinting is rarely permanent, but does involve some architectural changes in the CSK on the time scale of interest. The molecular underpinnings of polarity in yeast cells are reasonably well understood, and mathematical models have been developed to investigate the role of different pathways in the polarization process [111,112,113,114,115,116]. However, the underlying networks are more complex in motile cells such as Dicty and neutrophils, and progress has been slower.

While polarity at the molecular or CSK level can arise in the absence of external signals, direction-sensing involves the detection of spatial differences in an external signal over the cell surface, thereby determining what the most favorable direction is according to some criterion. This typically leads to the localization of some components in the membrane that can initiate structural polarization at the level of the CSK, or if the best direction of the signal changes, lead to either re-polarization of the cell or re-orientation of it. Typically, there is a threshold level of the signal needed to initiate a response, and this may vary depending on the history of exposure to the signal, since some cell types adapt to constant signal levels. In addition, many cell types can detect low levels of a signal, but then amplify them internally to initiate the appropriate intracellular response.

### 5.1. Mathematical Models for Polarization

#### 5.1.1. Localization of ‘Hotspots’ for Wave Initiation and Polarization

We have seen that a variety of membrane waves exist in Dicty and other cell types, but how they are initiated is not well understood. Mathematically speaking, it is known that different perturbations of an excitable system can lead to waves, but how the perturbation that triggers the event in question arises spontaneously in the membrane environment is not known. Here we describe two different mechanisms that may be involved—a spontaneous coagulation mechanism that creates a spatially distinct region or ‘hotspot’, and a positive feedback mechanism that has a similar effect. In either case the objective is to create a localized nanostructure in the membrane that initiates the appropriate activity. Important questions concern how large the nanostructure must be in spatial extent, how long it must persist in time to create observable events such as propagating waves, and how long its effects persist. In the case of PIP2-PIP3 waves the effect may be relatively short-lived, since once the wave has passed the system may relax to the unperturbed state, whereas in other cases it may persist over a much longer time scale.

Modeling of symmetry-breaking, which usually means establishment of polarity in a cell, addresses either the question of ‘long-term’ or imprinted polarization, as in the budding yeast or a cell migrating along the gradient of a signal, or relatively short-term polarization, either spontaneous or in response to a fluctuating extracellular signal. In persistent polarization the initial response to the event may stimulate reinforcing events, such as modification of the CSK that prolong the asymmetry or polarity for a longer period. This will be discussed in the context of direction-sensing in response to an external cu—here we discuss establishment of localized nanostructures.

**Figure 15:**
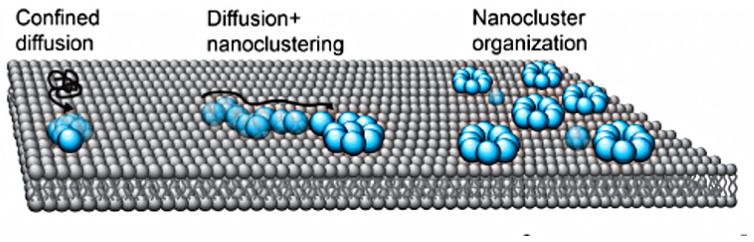
Formation of naonclusters on a lipid bilayer. From Sartorel et al. [117].

The general mechanism of cluster formation is illustrated in Figure 15, which shows how diffusion on the membrane can lead to clusters of proteins. The model arose in the context of cell polarization from the observation that surface-bound Cdc42 forms nanoclusters in the membrane in budding yeast [117]. The clusters diffuse more slowly than single molecules and are larger at the cell poles and thus they tend to localize there [118]. Clearly interactions with the membrane and other proteins must lead to a reduction in the free energy of the system, else there would be no clustering, and it is found that the cluster size depends on both the scaffold protein Bem1 and the lipid environment, in particular phosphatidylserine levels [118].

At sufficiently high densities the process can be described by the Smoluchowski equation

(2) $$\frac{\partial f_{n}}{\partial t}\left(x,t\right)=D\left(n\right)\Delta f_{n}\left(x,t\right)+Q_{1}^{n}\left(f\right)\left(x,t\right)-Q_{2}^{n}\left(f\right)\left(x,t\right).$$

Here $f_{n}$ is the membrane density of clusters of size *n*, D(n) is the diffusion coefficient of such clusters, and Δ is the Laplacian on the surface. The term

(3) $$Q_{1}^{n}\left(f\right)\left(x,t\right)=\frac{1}{2}\sum_{m=1}^{n-1}\alpha \left(m,n-m\right)f_{m}\left(x,t\right)f_{n-m}\left(x,t\right),$$

represents creation of clusters, where α is the coefficient of creation of a cluster of size *n* from clusters of size *m* and n−m. Similarly, the loss term is given by

(4) $$Q_{2}^{n}\left(f\right)\left(x,t\right)=f_{n}\left(x,t\right)\sum_{m=1}^{\infty }\beta \left(m,n\right)f_{m}\left(x,t\right).$$

Of course treating the aggregation process using continuum densities may not be valid in general, in which case one must revert to a stochastic simulation of Equation (2). This was done for general reaction-diffusion equations in Hu et al. [119] and in the context of aggregation on membranes in Turner et al. [120] and Richardson et al. [121].

(5) $$\frac{\partial u}{\partial t}\left(x,t\right)=\frac{1}{2}D\Delta u+k_{on}\left(1-h\right)+k_{fb}\left(1-h\right)u-k_{off}u,$$

**Figure 16:**
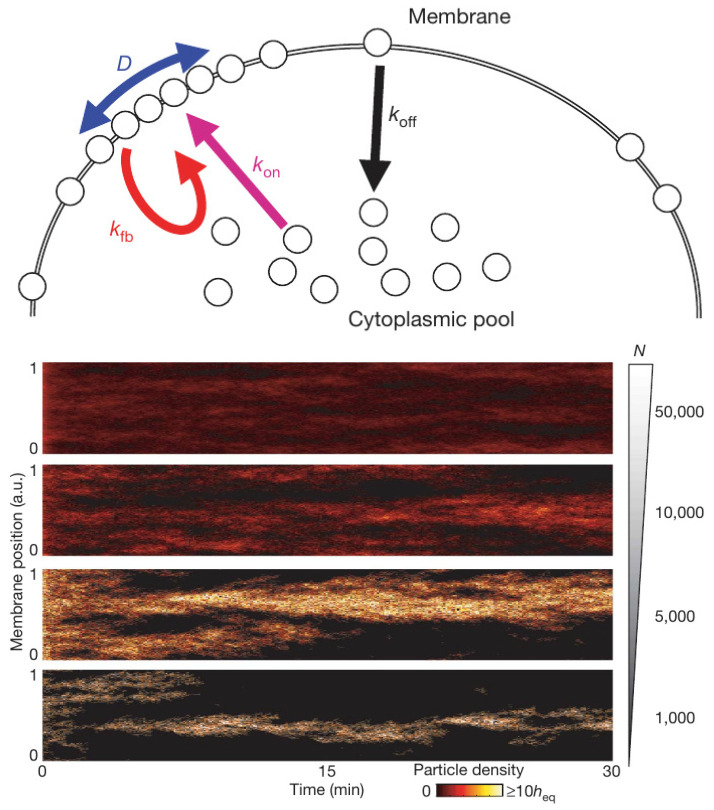
The reinforced-binding model. Modified from Altschuler et al. [122].

Another mechanism closely related to models described in the next section involves reinforcement of binding from the cytosol by previously bound ligands on the membrane. The simplest model of this involves a single species that is either bound to the membrane or is freely diffusing in the cytosol [122]. The molecular species shuttles between the cytosol and membrane in a simple on-off step, but the rate of binding to the membrane can also be increased by membrane-bound species, as shown in Figure 16 (top). The governing equation for such a reinforced-binding mechanism in which diffusion on the membrane is allowed is where *h* is the membrane-bound fraction of the total number of molecules—both in the cytosol and on the membrane [122].

Again the question of whether a continuum description is appropriate arises, and the authors tested a stochastic simulation of Equation (5) and found that the results depend strongly on the total number of particles, as shown in Figure 16 (bottom). When there are many particles the entire membrane is covered and the distribution of signaling molecules on the membrane converges to a homogeneous steady state. However, when there are few particles—1000 under the conditions used—the model with a positive feedback alone is sufficient to create and maintain a single localization site of membrane-bound molecules. This model has also been applied by Houk et al. [123] to explain how membrane tension maintains cell polarity by confining signals to the leading edge during neutrophil migration.

**Figure 17:**
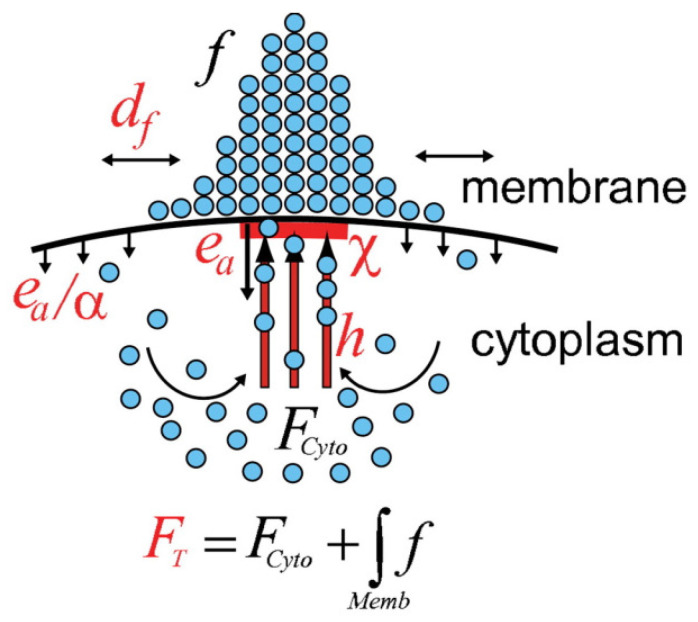
Schematic diagram of the model. From Marco et al. [124].

A different model for localization depicted in Figure 17 was proposed by Marco et al. [124]. In this model localization stems from the balance of three processes: diffusion along the membrane, transport to the membrane along actin or microtubules, and recycling to the cytoplasm via endocytic uptake and membrane recycling. In this model local polarization of the CSK is assumed to entail active transport, but the objective is to show how proteins can be localized on the membrane.

Let *f* denote the density distribution of the protein on the membrane, and let $F_{cyto}$ be the homogeneous cytoplasmic concentration of the protein. Then the evolution of *f* is governed by

(6) $$\frac{\partial f}{\partial t}=d_{f}\Delta f-\left(e_{a}\chi +\frac{e_{a}}{\alpha }\left(1-\chi \right)\right)f+hF_{cyto}\frac{\chi }{\int \chi },$$

where $d_{f}$ is the diffusion constant on the membrane and χ is the directed transport window function defining the region of the plasma membrane to which cytoskeletal tracks are attached. Furthermore, *h* is the directed transport rate along cytoskeletal tracks, $e_{a}$ is the endocytosis rate within the directed transport region, α is the ratio of the endocytosis rates within and away from the directed transport region, and $F_{cyto}$ is the cytoplasmic pool of *f* which is homogeneous due to the fast dispersion in the cytosol. Their experimental results corroborate the assumption that co-localization of endocytosis with actin patches in the polarized region is important for cell polarity. Of particular significance is the theoretical result that endocytosis rates can regulate dynamically balanced systems to optimize the asymmetric localization of membrane-protein distributions. Endocytosis will reappear later in the context of regulating the spatial distribution of WASP in Dicty.

Which of the three mechanisms might be involved in wave formation as described earlier? The reinforced-binding mechanism and the endocytosis model are directed more to creation of permanent ‘poles’ in a cell, rather than an ephemeral ‘hot spot’, since there is no explicit mechanism for deconstruction of the localization by turning off the positive feedback on binding, while localization in the cluster-formation model is more likely to be disrupted due to membrane fluctuations.

#### 5.1.2. Reaction-Diffusion Models for Gradient Establishment

In the clustering and reinforced-binding mechanisms polarity arises independently of the CSK, but when motility is involved bidirectional interaction of signaling and the CSK is essential, as seen in the model of phagocytosis. When there is a link the most complete model description involves both the signaling networks and the mechanical effects of membrane deformations and pseudopod growth, and there is as yet no mechanistic model of these interactions. At the other extreme, there are many mathematical models that have been formulated that contain no link between signaling and the CSK, and the objective in these is to establish a gradient of a signaling molecule involved in polarization. Meinhardt [125] suggested an activator-inhibitor model that incorporates a third species that functions as a local inhibitor. Small external differences are amplified via a Turing instability in the activator-inhibitor system, and the slower in-activator suppresses the primary activation. It was shown that transient maxima of the internal signals arise at random locations in the absence of external signals, and for suitable parameters the model can generate stable cell polarization. This model is an interesting high-level description of the process, but has no direct relation with the underlying biochemistry in any system.

A class of more recent models for gradient establishment and direction-sensing are also based on an activator-inhibitor mechanism. These so-called LEGI—local excitation and global inhibition—models are used to explain direction sensing and adaptation in a constant chemoattractant field [126]. The models incorporate a fast-responding but slowly diffusing activator and a slow-acting, rapidly diffusing inhibitor, similar to what is used in a Turing mechanism, to set up an internal gradient of activity that tracks the extracellular gradient. The usefulness of such models is limited because of the oversimplification of the signal-transduction network, and the need for a wide disparity in the diffusion coefficients of the inhibitor and activator to establish an intracellular gradient.

Other models have been built around two-component systems of reaction-diffusion equations that involve binding of a cytosolic species to the membrane, and in these the difference in diffusion rates arises from the fact that one component diffuses in the cytosol and the other on the membrane. Such models are typically described by a system of the form,

$$\begin{array}{ccc} \frac{\partial u}{\partial t} & = & D_{u}\Delta u+f\left(u,v\right)\\ \frac{\partial v}{\partial t} & = & D_{v}\Delta v+g\left(u,v\right). \end{array}$$

where *u* is the cytosolic concentration and *v* is the areal density on the membrane. It is assumed that *u* is constant in the direction normal to the surface, which is appropriate for a membrane under a thin layer of fluid, but not in general, and thus f(u,v) represents the binding step. Furthermore it is usually assumed that binding is the only reaction, and that f(u,v)=−g(u,v), i.e., *v* and *u* are membrane-bound and cytosolic forms of the same species that are converted point-wise in space. However, this is not strictly correct, since the volumetric change in *u* is not equivalent to the areal change in *v* [127].

In general, f(u,v) incorporates both a nonlinear feedback component that reflects reinforcement of binding, i.e., a form of autocatalytic binding, as in the model described earlier, as well as saturation. Otsuji et al. [128] have used several different forms of the nonlinearity, *viz.*,

(7) $$f\left(u,v\right)=a-1\left\{\frac{u+v}{{\left[a_{2}S\left(u+v\right)+1\right]}^{2}}-v\right\}\mathrm{or}f\left(u,v\right)=-a_{1}\left(u+v\right)\left[\left(\alpha u+v\right)\left(u+v\right)-a_{2}\right],$$

where $D_{u}=\alpha D_{v}$, *S* is intensity of stimulation, and $a_{1},a_{2}$ are model parameters whose biological meanings are obscure. In a model due to Mori et al. [129] *f* is chosen as

(8) $$f\left(u,v\right)=\left(k_{0}+\gamma \frac{u^{n}}{u_{0}^{n}+u^{n}}\right)v-\delta u,$$

where $k_{0}$ is the rate of activation of *u* from *v* and δ is the rate of inactivation of *u* to *v*. Here a Hill function in *u* alone, as distinct from the first form in Equation (8), was used to describe self-activation or binding of *u*. The authors show that a wave initiated at one boundary can stall or be ‘pinned’ under suitable conditions, thus leading to stable polarization.

**Figure 18:**
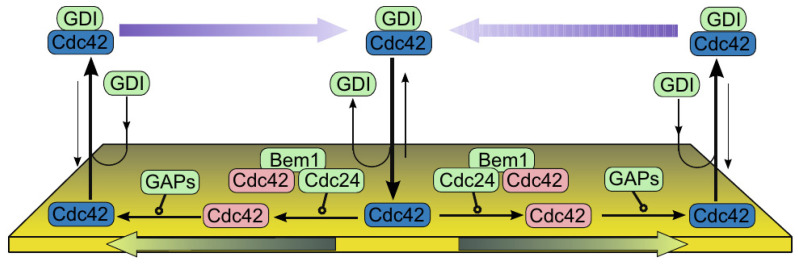
The components of a model for self-organized clustering of activated Cdc42 in yeast. Blue (pink) represents inactive (active) Cdc42 in the cluster. Inactive Cdc42 is activated at the membrane by a complex of active Cdc43, Bem1 and Cdc24. The guanine dissociation inhibitor (GDI) shuttles inactive Cdc42 to and from the membrane. From Goryachev and Pokhilko [111].

While these models provide some insight into cell polarization, they are in general too simplistic to make significant predictions concerning polarization in a given system. The most significant limitation of all models of the type at Equation (8) is that the stimulus is restricted to one point on the boundary. As a result, they cannot be used for understanding how a cell in reacts to a graded signal, since there is no extracellular signal except at the point of stimulation. A second problem concerns how the structure of the nonlinearities used might arise from a mechanistic description. Figure 18 shows the steps in a mechanistic description of yeast polarization, which would lead to a complex system of equations for components on the membrane that would be difficult to describe with two variables. Other models of the type in Equation (8) are reviewed in [130].

### 5.2. A Model for Direction-Sensing in Dictyostelium in cAMP Gradients

For a Dicty cell to align with the local gradient in a noisy chemotactic field, it must measure the local cAMP concentration at its surface and determine the direction in which to move. A precise choice is not necessary—a mathematical model predicts that cells can aggregate as long as they choose their direction within a cone of ±135° of the correct direction, but they aggregate more slowly [131]. A computational model of the $G_{\beta \gamma }$-AC-cAMP part of the network in Figure 5 shows that a for a sufficient length of time a cell experiences a significant difference in the front-to-back ratio of cAMP when a neighboring cell signals [132]. It follows from this that other components in the signal-transduction pathway will sustain similar front-to-back differences in a gradient, and experiments have shown that this holds for PIP3, PI3K, and PTEN.

A more recent model for the first downstream steps in the signal-transduction pathway in Dicty incorporates more of the underlying biochemistry and can replicate a number of experimental observations. These include amplification at the level of RasG (hereafter simply Ras), the observed biphasic response to graded stimuli, the existence of a refractory period for repeated stimuli, and ‘memory’ of the up-gradient direction in a wave [133]. In LatA-treated cells [134,135] the feedback effect from the actin cytoskeleton on Ras is eliminated, and the model is based on these experiments. Figure 5 shows that activated Ras activates PI3K and other downstream steps to actin polymerization, but the model was restricted to the Ras dynamics in response to cAMP because there is no known direct feedback to Ras from downstream steps between Ras and the actin cytoskeleton.

**Figure 19:**
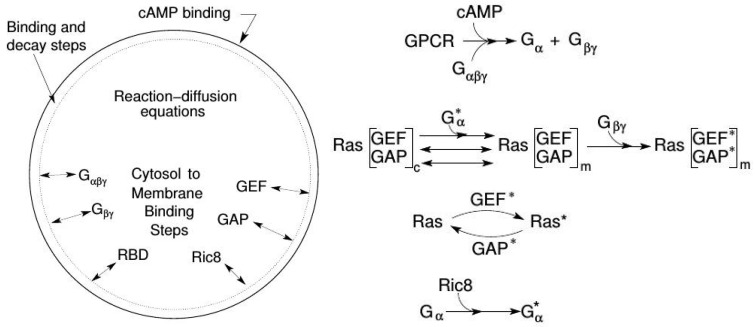
A schematic of the major processes in the model (**left**), and the primary steps in the network (**right**). From Cheng and Othmer [133].

The model involves three main processes: signal detection via CAR1, transduction based on activation of $G_{\alpha_{2}\beta \gamma }$, and activation of Ras (Figure 19). The key components in the model are $G_{\alpha_{2}\beta \gamma }$, Ric8, (a GEF that activates $G_{\alpha_{2}}$ [136]), Ras, and RasGEF and RasGAP. All components except $G_{\alpha_{2}}$ cycle between the membrane and the cytosol. RasGEF and RasGAP are activated at the membrane by free $G_{\beta \gamma }$, and the translocation of RasGEF from the cytosol is enhanced by the activated form of $G_{\alpha_{2}}$.

It has been observed [134] that adaptation to constant cAMP stimuli occurs at the level of Ras, whose activity is controlled by a balance between RasGEF* and RasGAP*—none of the upstream components adapt. At low stimuli adaptation is near perfect, but at higher stimuli adaptation is imperfect. The model is able to capture the dose-dependent Ras activation and various patterns such rectification and refractoriness under uniform stimuli. It can be shown that $G_{\alpha 2}^{*}$ contributes to the observed imperfect adaptation in a uniform stimulus due to the asymmetrical translocation of RasGEF. Earlier we noted that the cell-level response to a uniform stimulus is a ‘cringe’, which appears within about 20 s and lasts about 30 s—comparable to the time-scale for adaptation of the Ras response.

Another experimental observation under uniform stimuli is that cells exhibit a refractory period after stimulation [10]. A short delay following a stimulus leads to a small response, and the response increases if the delay is increased. This is often taken as an indicator of excitability, but there is no indication that there is a threshold stimulus in the experiments or the model—the maximum Ras* response increases monotonically over four orders of magnitude of the stimulus. This can be explained by considering the ratio RasGEF*/RasGAP*. For short delays the slower inactivation of RasGAP* reduces the amount of Ras that can be activated. Under uniform stimuli Ric8 plays a minor role and *Ric8-null* cells respond essentially as WT cells. However, it plays a major role under graded stimuli.

**Figure 20:**
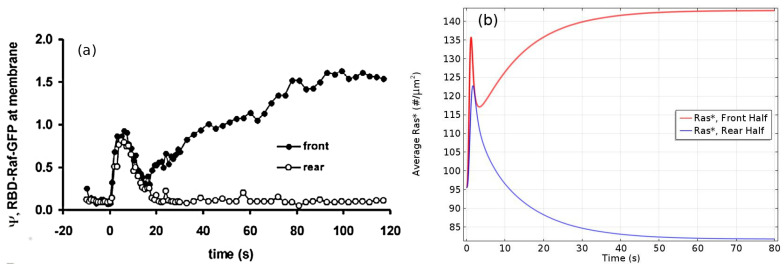
The levels of activated Ras* at the front and back in a static cAMP gradient as a function of time measured experimentally (**a**) From Kortholt et al. [135] and the model prediction (**b**) [133] From Cheng and Othmer [133].

Under graded stimuli the response in LatA-treated cells is biphasic: on a short time-scale (10 s) Ras is activated over the entire membrane, the activation decays within 20 s, and this is followed by a persistent polarization of Ras activation that is high at the high point of the gradient. The model reveals that the fast time-scale of $G_{\beta \gamma }$ mediated RasGEF and RasGAP activation induces the first transient Ras activation on the entire membrane, while the slow time-scale of overall equilibration—which includes redistribution due to diffusion, membrane localization and positive feedback between Ric8 and $G_{\alpha }$—induces the delayed secondary response that produces the symmetry breaking. Figure 20a,b show that the biphasic response—initially uniform around the cell, followed by symmetry-breaking later—in a graded stimulus is captured.

**Figure 21:**
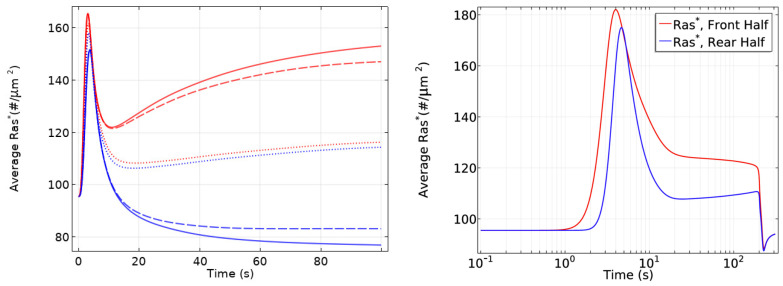
(**Left**) The effect of diffusion on the biphasic response. Solid, dashed and dotted lines are for D = 0, 0.1 and 10 μ$m^{2}$/s resp.—cytosolic diffusion of all components: D = 30 μ$m^{2}$/s. (**Right**) The average Ras* in the front and rear halves in response to a passing triangle wave. From Cheng and Othmer [133].

Important insights into the role of diffusion emerge from the model. While all cytosolic components diffuse at the same rate, the model predicts the observed symmetry-breaking, and analysis shows that diffusion of both RasGEF, the activator, and RasGAP, the inhibitor, is necessary. Simulations also show that there is no symmetry-breaking in $g_{\alpha }$-null cells and there is no direction-sensing in Ric8-null cells when exposed to a shallow gradient or a steep gradient with high mean concentration. Finally, slow diffusion of components on the membrane enhances, but is not necessary, for symmetry-breaking. As shown in Figure 21 left, only unrealistically high diffusion rates on the membrane removes the biphasic response. In particular, symmetry-breaking does not require a disparity between the diffusion coefficients of the activator (RasGEF) and the inhibitor (RasGAP), as is required in LEGI models.

**Figure 22:**
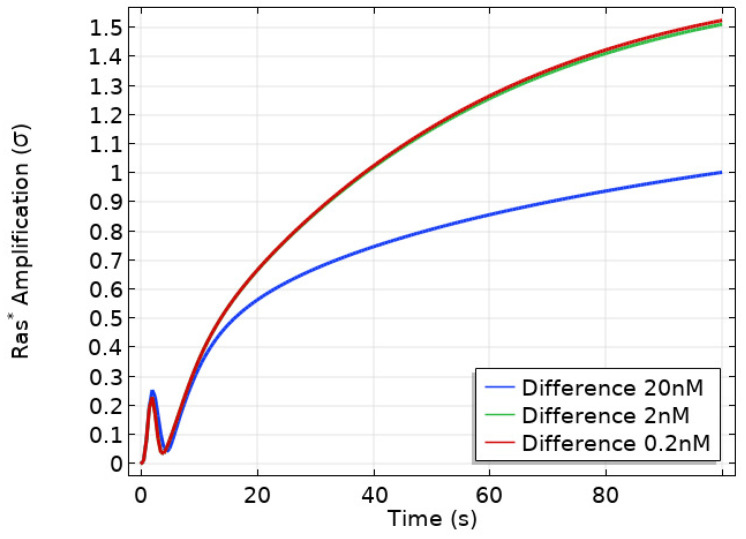
Amplification, as defined in [133], of the Ras signal in an unpolarized cell in a linear gradient with mean 10 nM and front-to-back differences as labeled.

Well-polarized cells are able to detect and respond to chemoattractant gradients with a 2% concentration difference between the anterior and posterior of the cell [63], and in Figure 22 we show the results of the model predictions for the response to the Ras gradient in unpolarized cells. One sees that the amplification is significant for a 2% and 20% difference, but less for a very steep gradient. Amplification of a cAMP gradient stems from two outputs of the network. First, the $G_{\alpha 2}^{*}$ concentration on the membrane is highest where the cAMP concentration is highest, and this produces higher localization and activation of Ric8, which reactivates $G_{\alpha 2}$ and further promotes RasGEF localization there. Secondly, faster $G_{\alpha_{2}\beta \gamma }$ re-association at the rear because Ric8 is lower there, which leads to lower $G_{\alpha 2}^{*}$ and creates gradients of $G_{\alpha_{2}\beta \gamma }$ and $G_{\beta \gamma }$, the former high at the rear and low at the front, and conversely for the latter, as is observed experimentally [137]. Furthermore, Ric8 contributes to the amplification of Ras activity by regulating $G_{\alpha 2}$ dynamics: the reactivation of $G_{\alpha 2}$ by Ric8 induces further asymmetry in $G_{\alpha_{2}\beta \gamma }$ dissociation, which in turn amplifies the Ras activity. Thus $G_{\alpha_{2}\beta \gamma }$ cycling modulated by Ric8 drives multiple phases of Ras activation and leads to direction-sensing and signal amplification in cAMP gradients. The biphasic response can be understood as follows. Initially the cAMP stimulus produces a nearly uniform response due to rapid diffusion of $G_{\beta \gamma }$ in the cytosol, but on a slower time-scale symmetry-breaking is driven by an ‘indirect’ positive feedback between Ric8 and $G_{\alpha_{2}}$ (activated $G_{\alpha_{2}}$ promotes Ric8 binding at the membrane, and activated Ric8 promotes reactivation of $G_{\alpha_{2}}$). Increasing diffusion of membrane components reduces the spatial asymmetry this produces.

In an imposed triangular wave of height 1 μm and wavelength 1 mm [138], Ras* at the front is always larger than at the rear throughout passage of the wave (Figure 21 right), which reflects a form of ‘memory’ of the point at which the cell first received the signal. This shows that symmetry-breaking at the level of Ras encodes sufficient ‘memory’ to maintain directional orientation during a passing wave and thus provides a solution to the ‘back-of-the-wave’ problem, in that cells do not turn to follow the cAMP gradient after the wave has passed, despite the fact that the spatial gradient reverses as the wave passes over the cell [10,139]. It should be emphasized that the model was built on the rounded LatA-treated cells that have no intrinsic polarity, which suggests that polarity is not necessary for the persistence of direction-sensing at the natural wave speed, even at the level of Ras activity.

## 6. The Integration of Signaling, Polarization and Structural Changes in the CSK

In general, establishment of polarity in an un-stimulated, unpolarized eukaryotic cell at rest that is exposed to a graded, time-independent external signal involves three major steps.

1. Detection of the chemical and mechanical signals in the ME with membrane receptors, adhesive sites, and other detection mechanisms.
2. Transduction of the extracellular signals into spatially biased intracellular signals that reflect the external signals and activate one or more downstream signaling pathways.
3. Translation of the output of these signaling pathways into the changes in the CSK needed to begin directed motion.

Similar steps occur in an already-polarized cell, but in that case the last step also involves the decision to change direction if necessary, or to simply continue motion.

While these steps may appear to involve simple feed-forward processes, there are numerous feedback loops between the signaling pathways (Figure 5) and significant overlap in their downstream effects, and thus the balances between them determine the response when all are functional. The complexity of the CSK [140] and the fact that the same cell type can use very different modes of motion in different MEs makes it difficult to translate what is known about steps 1 and 2 into a set of ‘rules’ for carrying out step 3. Moreover, we have thus far focused on chemical signals to the exclusion of mechanical signals, but Dicty, neutrophils and other cell types continuously monitor their ME and adapt their mode of movement to it. For example, in a fluid Dicty swims, while in other environments it moves either by extending pseudopods and contracting the rear, by blebbing, or by a combination of these. The evolutionary advantage of this flexibility is clear, but it also means that determining the rules for implementing step 3 remains a major challenge. However, we can identify components of what is involved in implementing step 3 under chemotactic gradients, which is done in the context of Dicty next. Moreover, the component parts are fairly universal [4], and there is evidence that mechanical stimuli act through the same pathways as chemical signals in Dicty [141].

### 6.1. How Graded Chemical Signals Lead to Polarization

Since cAMP receptors remain uniformly distributed on the membrane following stimulation [137], polarization first occurs at the level of Ras, $G_{\alpha_{2}}$, and $G_{\beta \gamma }$ (Figure 5), followed by adaptation in Ras activation. Activated Ras activates PI3K, which leads to a local increase in PIP3 production and a local increase in PI3K, the latter dependent on actin polymerization [86]. Thus, without any interaction with other pathways, there is a front-to-rear (Hereafter we refer to the region on the membrane that receives the highest stimulus as the ‘front’, and the antipodal part the ‘rear’). decrease in activated Ras, PI3K and PIP3. Since all points on the cell receive the cAMP signal, the signal transduction network is active over the entire cell and the gradients that arise are the global composite of local changes and diffusive and other types of transport.

PIP3 has a PH domain that serves as a docking site for cytoplasmic proteins such as the the GTPase Rac1 and the kinase Akt. The increase in PIP3 leads to rapid binding and activation of Rac1 via a GEF, and rapid localization and activation of Akt, which is essential for CSK polarization and chemotaxis—mutants lacking Akt cannot polarize the CSK properly in a chemotactic gradient and the cells move slowly [142]. Experimentally it is found that rapid withdrawal of the gradient leads to the return of PTEN and ${\mathrm{PH}}_{CRAC}$-GFP (labeled CRAC) to their pre-stimulus distribution, but reapplication of a uniform cAMP stimulation produces a clear ${\mathrm{PH}}_{CRAC}$-GFP translocation to the rear, but not to the front [143]. This indicates that a stronger ‘inhibition’ of polarization is maintained at the front of a polarized cell. It was shown that this inhibition is not caused by PTEN, G$\alpha_{1}$ or G$\alpha_{9}$, but the observations remain to be explained.

The SCAR/WAVE regulatory complex (WRC) is a five-protein complex that binds both activated Rac1 (Rac1GTP) and Arp2/3, and thus provides a link between the two that leads to formation of branched actin [144]. Another member of the WAVE family, WASP, also binds Rac1GTP, and can activate Arp2/3 and produce pseudopods in the absence of WAVE, but plays other roles when WAVE is expressed [145]. The protein complex DGap1/cortexillin also binds Rac1GTP, but apparently only acts to sequester it [146]. Cortexillin is known to bind to PIP2, which increases down-regulation of Rac1 at the rear.

Because PTEN docks to PIP2, the reduction of PIP2 due to conversion into PIP3, coupled with possible inhibition of PTEN localization by PIP3 [147], reduces the membrane-attached PTEN, which produces a reverse gradient in bound PTEN and further increases PIP3 at the leading edge. In addition, an increase of PTEN at the rear decreases PIP3 there, further amplifying the front-to-rear PIP3 gradient. Thus, one of the second steps in polarization is establishment of the front-to-rear gradients in PIP3, AKT, and the SCAR/WAVE regulatory complex (WRC) and the reverse gradient in PTEN.

Myo-II has several effects in the cortex. One is to stabilize it by associating with anti-parallel linear actin filaments to produce actomyosin, and the other is contraction of the filaments needed both in movement by blebbing and via pseudopods. The motor activity of myo-II, independent from its cross-linking function, is up-regulated by myosin light chain kinases (MLCK). In a pathway parallel to the $G_{\beta \gamma }$ pathways, $G_{\alpha_{2}}$ activates Rap1 (Figure 5a) and a downstream effector, the kinase Phg2. This localizes and activates the heavy-chain kinase MHCK, which leads to myo-II disassembly [148] and in turn reduces the cortical density and facilitates branched actin polymerization and pseudopod extension. PakA inhibits the cGMP-promoted MLCK activation of motor activity and hence reduces contractility [43], and together this leads to a front-to-back gradient of free myo-II, which can lead to an increase of its L-actin-binding at the rear. Thus, the spatio-temporal balance of the effectors of the cGMP, Ras and Rap1 pathways controls actin polymerization and actomyosin assembly, as well as their spatial localization [4].

It is known that myo-II is localized at the rear of migrating Dicty cells [149], but whether PTEN controls its localization is not known. It has been shown that PTEN localization at the sides and the rear of cells occurs prior to myo-II localization there [150], and it was suggested that PTEN may be involved in a positive feedback loop in which contraction enhances accumulation of PTEN and myo-II [150]. Since PI(4,5)P2 promotes membrane-binding of PTEN, the gradient of PIP2 increases its posterior localization [151], but PTEN is not the sole controller of myo-II localization—it still localizes in ${pten}^{-}$ cells. This may involve the cGMP pathway in Dicty [152,153], and in other cells myo-II preferentially binds to actin filaments in tension, and a reduction in the tension leads to release of myo-II [154].

In the presence of diffusion of components on the membrane and in the cytosol, the composite effect of the processes described would be to produce smooth variation of the components on the membrane and those in the cytosol. If we define ‘frontness’ by a propensity to produce predominately branched actin and pseudopods, whereas ‘rearness’ is characterized by a preponderance of linear actin and actomyosin, how does a cell polarize into a well-defined front and rear? Do these characteristics vary smoothly in proportion to the gradients described above, or are there additional steps that sharpen the distributions? Experimental images of tagged components suggest the latter, but this can be misleading because there is always a threshold in detection of labeled components. Assuming that the separation is quite sharp, how can the gradients be amplified locally? For instance, if there is cooperative binding similar to that in models described earlier, will the frontness and rearness be more clearly separated? Since activated Rac1GTP and WAVE are key components in branched actin production, can the WRC and Rac1GTP be localized more sharply at the front?

**Figure 23:**
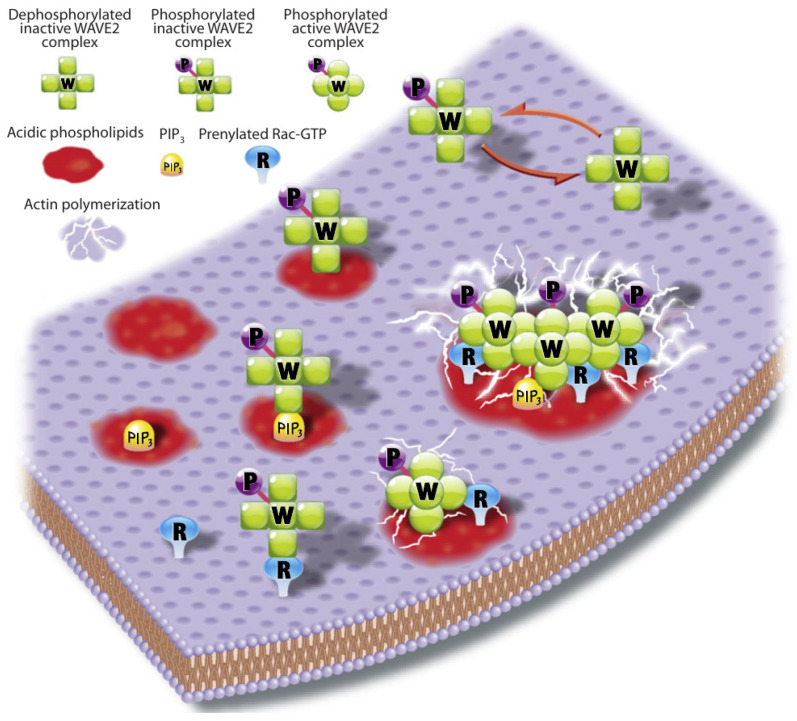
A possible step in the localization of WRC. From Lebensohn and Mitchison [144] with permission.

WAVE binds to both Rac1GTP and PIP3, and a possible step in this direction is shown in Figure 23, where the WRC-Rac1GTP-PIP3 units form clusters that produce branched actin more rapidly than the sum of the individual units. If complex formation between WRC-Rac1GTP and PIP3 evolves according to

(9) $$\frac{\mathrm{dC}}{\mathrm{dt}}=F(\mathrm{WRC}-\mathrm{Rac}1\mathrm{GTP},\mathrm{PIP}3)\cdot \mathrm{WRC}-\mathrm{kC}$$

where C is the WRC-Rac1GTP-PIP3 complex, then localized C will result for an appropriate F provided diffusion in the membrane is not too rapid. For instance, if F is increasing in both arguments and reflects cooperativity in Rac1GTP and PIP3, either separately or jointly, then formation of the complex will be restricted to regions in which both Rac1GTP and PIP3 are large. This is not a mechanistic description, but rather a qualitative argument of what a more detailed mechanism could produce. Moreover, this is not the complete story in Dicty, for in the absence of WASP, WRC-RacGTP accumulates at the rear of the cell [155]. In addition to activating Arp2/3, WASP is also thought to remove Rac1GTP from the membrane, thus depleting active Rac1GTP at the rear. DGap1/cortexillin complexes may have a similar role. The combination of these steps can lead to a relatively sharp variation between the region in which formation of branched actin dominates and that in which linear actin and actomyosin prevail.

An alternate approach to generating the separation between frontness and rearness has been suggested in the context of cancer cells [156]. In that approach a sharp demarcation is achieved with a network in which RhoA and Rac1 are linked by a double-negative feedback loop. This leads to a spatial distribution of RhoA, Rac, and the inhibitor PAK, and the boundary between frontness and rearness occurs at particular values of PAK at which there is a spatial discontinuity in the RHoA and Rac distributions. Such discontinuities would be difficult to sustain in the presence of diffusion, but the effects of diffusion are not considered by the authors.

Other factors may also play a role in polarization. For example, cofilin promotes breakup of actin filaments, and suppression of its expression results in re-localization of Arp2/3 to one pole and protrusions from only that pole [157]. Myo-IB, the membrane-cortex linker protein [81], preferentially binds to PIP2, and thus is released when PIP2 is converted to PIP3. Another potential factor is profilin, which increases formin-mediated elongation rates in a concentration-dependent manner [158]. At the same time, profilin-bound monomers inhibit the polymerase activity of WH domains of SCAR/WAVE and WASP by competing for G-actin monomers [159]. Although recent studies demonstrate that WAVE contains a proline-rich domain, which is capable of delivering free actin monomers to barbed ends in vitro [160], its activity could be slow compared to that of filament elongators such as formins.

### 6.2. The Role of Membrane and Cortical Tension in Polarization

**Figure 24:**
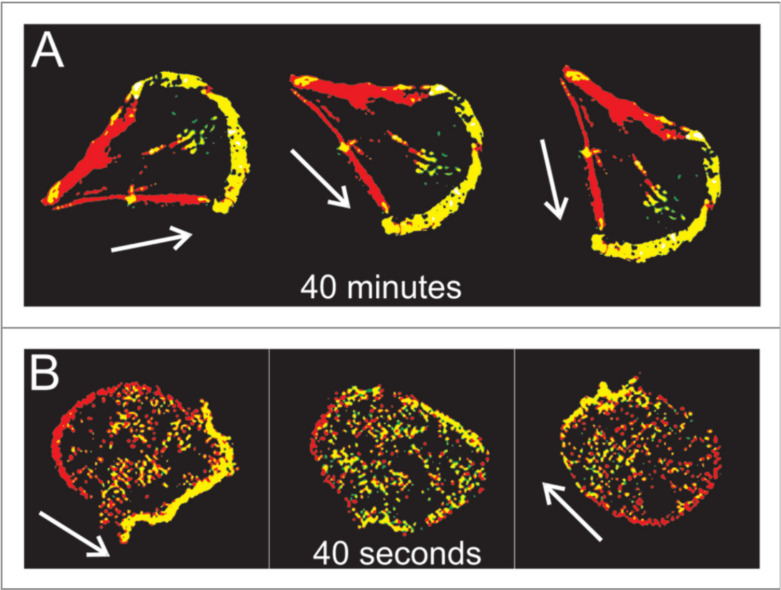
Re-orientation of fibroblasts (**A**) and Dicty (**B**). From Faix et al. [162].

A question that arises in the context of the preceding models in which diffusion is the primary transport mechanism is whether diffusion is fast enough to change the polarity of a cell in response to changes in the signal. In Dicty directional changes of a shallow gradient induce polarized cells to turn, whereas large changes lead to large-scale disassembly of motile components and creation of a new ‘leading edge’ directed toward the stimulus [161]. In Figure 24 one sees that fibroblasts require 40 min to re-orient 90°, whereas a Dicty cell can re-polarize in 40 s. Computational experiments based on the model in Section 5.2 show that Ras activation can be reversed in 50–60 s in response to large-amplitude reversals of the cAMP gradient, but diffusion alone may not suffice, since reversal becomes much slower when exposed to a weaker reversed gradient. Moreover, it has not been demonstrated that the necessary rearrangements of factors controlling the CSK can redistribute rapidly enough via diffusion. In fact, it has been shown that a diffusion-based polarization mechanism cannot provide long-range inhibition of secondary pseudopods in neutrophils, and it was suggested that membrane tension may be involved [123]. Since the membrane is generally modeled as elastic or viscoelastic, changes in tension propagate much more rapidly than diffusion-propagated signals, and may be involved in suppression of pseudopods toward the rear in both for Dicty and neutrophils.

Membrane tension plays a role in other contexts as well. Numerous proteins that contain a BAR domain can associate with curved membranes because they are sensitive to curvature [163]. Elevated membrane tension reduces the local curvature and can reduce the binding of such proteins [164]. This might regulate the membrane-binding of GEFs and GAPs that regulate the GTPase switches, which in turn provides feedback between curvature and actin dynamics. Cells such as fibroblasts sense the rigidity of the ECM via stress transmitted through integrin-mediated focal adhesions, which can lead to conformational changes in proteins within the complex. For example, in the case of BCAR1 proteins, force applied to the adhesion complex leads to exposure of phosphorylation sites for SRC-family kinases that can recruit signaling proteins and up-regulate the activity of Rac1 and Rap1.

In another example of mechanical effects, Dicty cells in a fluid flow establish a protruding front directed against the flow and a retracting rear, as indicated by labels for polymerized actin and myo-II markers at the front and rear, resp [165]. At a shear stress of ∼2.1 Pa the cell becomes polarized with an actin-enriched front upstream, and when the flow is reversed quickly, cells reverse their polarity in several phases. First, actin disassembles at the previous front between 0∼60 s after flow reversal. Then polymerization of a new front upstream begins at 30 s and stabilizes by ∼90 s. In the interim the amount of actin in the cortex decreases, which means that polarity reversal entails a significant re-building of the entire cortex. How shear stress is transduced into control of actin polymerization is not known, but as remarked earlier, it is thought that the signaling pathways are the same for both chemical and mechanical signals. Interestingly, the authors noted that similar patterns of front and rear inter-conversion were observed in cells re-orienting in strong gradients of cAMP.

**Figure 25:**
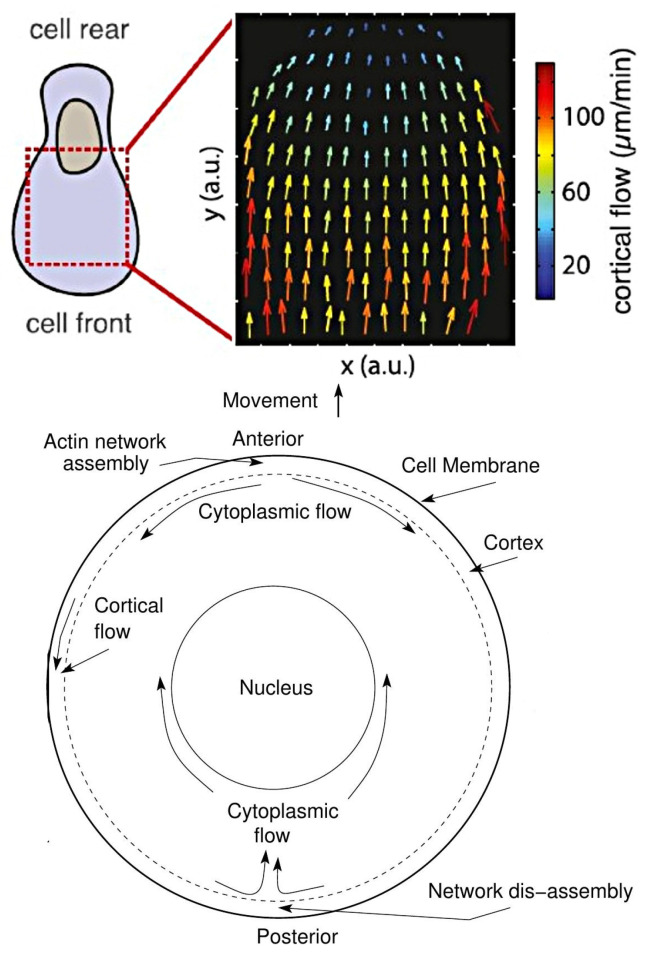
The measured cortical flow (**top**) (From Ruprecht et al. [166]), and the postulated intracellular flows (**bottom**).

Recent work has also shown that some cell types use strong cortical flows to propel themselves, and the intracellular actin flows that are generated polarize the cell and could move other signaling molecules axially. Ruprecht et al. [166] show that a stable non-polarized blebbing cell can be converted into a permanently polarized shape by increasing the contractility in cells. They also report cortical flow rates of 10’s of μms/min (Figure 25), which would induce an anterior-to-posterior cytoplasmic flow near the cortex, and thus a posterior-to-anterior flow in the center, as shown in Figure 25. The authors suggest that there is a high growth rate of the cortex at the front of a cell and a high disassembly rate at the rear, which would require a very different set of controls for the actin network.

A second stable-bleb type is more cylindrical and has a large uropod [167]. This also involves high myo-II activity and strong retrograde actin flow, and arises when slow mesenchymal cells undergo a MAT under low adhesion and confinement between plates. Evidence for involvement of the cortex in both cases is the fact that blebbistatin, an inhibitor of myo-II contractility, and LatA both inhibit polarization. It is thought that a gradient of cortical density and myo-II generates both the cortical flow and an axial pressure gradient in both morphologies, but what initiates the flow remains undetermined [168]. Computations reported in Wu et al. [17] show that tension gradients in the cortex can generate large-scale flows sufficient to carry monomers anteriorly, and thus can provide another mechanism for polarization by segregating components via the flow.

## 7. Epilogue and Open Problems

Our objective in this review was to describe some of the wide range of problems that arise in trying to understand cell motility. In the previous sections we discussed recent advances in understanding the dynamics of intracellular biochemical networks and how they are involved in actin waves, direction-sensing and polarization, but the problem of understanding how chemical and mechanical signals are used to control movement is both broader and deeper. Broader in the sense that other pathways not touched upon are involved, and deeper in the sense that our knowledge of the details of transduction of mechanical signals is shallow in many respects. As a result, there are many open questions that remain to be solved. Several that are closely related to topics discussed earlier are as follows.

- What is the minimal set of components of the network shown in Figure 5 that can control the random initiation of intracellular waves in un-stimulated cells? Experimental work described earlier suggests that a minimal set in Dicty may be SCAR/WAVE, Arp 2/3 and actin-binding proteins, but there are presently no models that can replicate the experimental results. A related question is what controls the initiation sites for pseudopodia. Is it randomness in the wave generation, or are there randomly located sites of decreased membrane tension that facilitate membrane deformation, or both?
- A question raised earlier concerns how cells establish a sharp demarcation between ‘frontness’ and ‘backness’ in the presence of an extracellular signal. This involves the spatial distribution of numerous species, and a minimal set of components to produce the demarcation is not yet known. A related question is how the strength of the signal determines whether the cell turns in response to a change in direction of the signal, or whether it completely rebuilds the CSK.
- There are as yet no models that integrate mechanical and chemical pathways to predict actin flows and structural changes in the CSK—even within a fixed cell shape. In the previous sections we simply described how some of the separate components may be involved in polarization, but their integration remains to be addressed.
- A larger question is how these pathways control the mode of migration used by a cell. Cells moving on flat surfaces often use lamellipodia, but movement in confined spaces can prevent the extension of lateral membrane protrusions, which may account in part for the use of blebs in confined spaces. The coexistence of blebs and pseudopods in Dicty suggests that the balance can be subtle, but there are experimental conditions under which one or the other dominates. Since cells often move in a spatially variable environment, the feedback from the ME can affect the mode of movement dynamically, and far more work is needed to understand how the cell-ME interaction controls the mode of movement. Significant progress has been made on simpler systems such as keratocytes moving on a flat surface [169], and recent techniques that can capture more dynamic shape changes in 3D via interface tracking shows promise [170], but much remains to be done. In the context of swimmers such as shown in Figure 3, a model in which protrusions propagate along the body length can replicate swimming speeds under various conditions [28], but how extension of protrusions is controlled by local fluid properties and other factors is not yet known.

Concerning pathways not described, the protein-calcium pathway in Figure 5 has attracted much less attention than other pathways in the context of chemotaxis, but it may play a significant role there. Early work suggested that calcium is not essential for chemotaxis [171], but other work shows that it plays an important role. Lusche et al. [172] show that extracellular calcium acts as a chemoattractant in parallel with cAMP, while other research shows that calcium is vital in cell migration of various cell types [173,174,175,176,177,178]. In macrophages and glial cells calcium influx plays a major role in maintaining the structure of the leading edge during migration [175,179]. In Dicty cAMP both stimulates and inhibits PLC activity via $G_{\alpha_{2}}$ and $G_{\alpha_{1}}$ protein subunits, resp. [180,181]. Kortholt et al. [182] reported that plc-null cells are resistant to the PI3K inhibitor LY294002 and produce little PIP3 after cAMP stimulation, while PLC over-expression increases PIP3, which affects chemotaxis similar to loss of PTEN. The dynamics of intracellular calcium range from individual stochastic events to global phenomena like waves and oscillations following stimulation [183,184,185], and given the excitability of the IP3-Calcium module, PIP2-PLC-Calcium and PIP3-PI3K-PTEN triangles can potentially inherit the excitability. Thus, an open problem is to investigate whether integration of the PIP2-PLC-Calcium triangle with the PIP2-PIP3-PI3K triangle could shed more light on the self-organization mechanisms.

Another aspect that deserves more attention concerns the role of stochastic fluctuations at various steps of the signaling pathways and network dynamics. Estimates made earlier of the signal noise in cAMP receptors in Dicty shows that noise may be important at low signal levels [47], but stochastic simulations of the exterior reaction-diffusion system are needed to make this more precise. Separately, given the more detailed models of intracellular signaling that are now available, an analysis of how cells cope with noise in the signals is feasible. For example, it was noted earlier that Dicty cells need not be very precise in their detection of the chemotactic gradient to aggregate, but imprecision carries the cost of less efficient aggregation [132]. Related to the question of how the random initiation of intracellular waves is controlled is the question of stochastic effects on the location of actin puncti at potential sites of protrusion. A stochastic model using a simplified signaling network shows how random actin spots can shrink and die or develop into full-fledged propagating waves [186], but further work on this is needed.

In summary, it is safe to say that a deep understanding of how the nanomachines that we call cells move is still is the future.

## Abbreviations

The following abbreviations are used in this manuscript:

F-actin	Branched and linear actin or either
CON	CSK oscillatory network
Dicty	Dictyostelium discoideum
$G_{\beta }\gamma$	G beta-gamma complex
$G_{\alpha 2}$	G-protein alpha-subunit
GEF	GTP exchange factor
GAP	GTPase-activating factor
DGAP1	IQGAP-related protein
PHP	PH domain proteins
Akt	PI3 kinase and protein kinase B
WRC	SCAR/WAVE regulatory complex
SHIP	SH2-containing inositol 5-phosphatase
WAVE	WASP-family verprolin-homologous protein
WASP	Wiskott–Aldrich syndrome protein
Arp2/3	actin-related protein 2 and 3 complex
AC	adenylate cyclase
B-actin	branched actin
${\mathrm{Ca}}^{2}$	calcium
CaM	calmodulin
CapP	capping proteins
Ctx	cortexilin
CAR	cyclic AMP receptor
cGMP	cyclic GMP
cAMP	cyclic AMP
CSK	cytoskeleton
CRAC	cytosolic regulator of adenylyl cyclase
DAG	diacylglycerol
ELMO	eukaryotic engulfment and cell motility proteins
ECM	extracellular matrix
G-actin	free actin monomer
GFP	green-fluorescent-protein
GDI	guanine dissociation inhibitor
GDP	guanosine diphosphate
GTP	guanosine triphosphate
GC	guanylate cyclase
IP3	inositol 1,4,5-trisphosphate
LatA	latrunculin A
L-actin	linear actin
LEGI	local excitation and global inhibition
MAT	mesenchymal-to-amoeboid transition
ME	microenvironment
MHCK	myosin heavy-chain kinase
MLCK	myosin light chain kinase
myo-IB	myosin-IB
myo-II	non-muscle myosin-II
PakA	p21-activated kinase A
PTEN	phosphatase and tensin homologue
PIP3	phosphatidylinositol 3,4,5-trisphosphate
PI3K	phosphatidylinositol-3 kinase
PIP2	phosphatidylinositol-4,5-diphosphate
PI5K	phosphatidylinositol-5 kinase
PLA2	phospholipase $A_{2}$
PLC	phospholipase C
PH	pleckstrin homology
STEN	signal-transduction excitable network
TORC2	target of rapamycin complex 2
WT	wild-type
